# Personalized interventions for behaviour change: A scoping review of just‐in‐time adaptive interventions

**DOI:** 10.1111/bjhp.12766

**Published:** 2024-11-14

**Authors:** Ting‐Chen Chloe Hsu, Pauline Whelan, Julie Gandrup, Christopher J. Armitage, Lis Cordingley, John McBeth

**Affiliations:** ^1^ Centre for Musculoskeletal Research University of Manchester Manchester UK; ^2^ Centre for Health Informatics, Division of Informatics, Imaging & Data Sciences University of Manchester Manchester UK; ^3^ Manchester Centre for Health Psychology University of Manchester Manchester UK; ^4^ NIHR Greater Manchester Patient Safety Research Collaboration University of Manchester Manchester UK; ^5^ The NIHR Manchester Musculoskeletal Biomedical Research Unit Central Manchester University Hospitals NHS Foundation Trust Manchester UK; ^6^ School of Primary Care, Population Sciences and Medical Education University of Southampton Southampton UK; ^7^ Present address: UCB Pharma UK Slough UK

**Keywords:** behaviour change, digital health, just‐in‐time adaptive intervention, mobile health, personalized intervention

## Abstract

**Purpose:**

Examine the development, implementation and evaluation of just‐in‐time adaptive interventions (JITAIs) in behaviour change and evaluate the quality of intervention reporting.

**Methods:**

A scoping review of JITAIs incorporating mobile health (mHealth) technologies to improve health‐related behaviours in adults. We searched MEDLINE, Embase and PsycINFO using terms related to JITAIs, mHealth, behaviour change and intervention methodology. Narrative analysis assessed theoretical foundations, real‐time data capturing and processing methods, outcome evaluation and summarized JITAI efficacy. Quality of intervention reporting was assessed using the template for intervention description and replication (TIDieR) checklist.

**Results:**

Sixty‐two JITAIs across physical activity, sedentary behaviour, dietary behaviour, substance use, sexual behaviour, fluid intake, treatment adherence, social skills, gambling behaviour and self‐management skills were included. The majority (71%) aimed to evaluate feasibility, acceptability and/or usability. Supporting evidence for JITAI development was identified in 46 studies, with 67% applying this to develop tailored intervention content. Over half (55%) relied solely on self‐reported data for tailoring, and 13 studies used only passive monitoring data. While data processing methods were commonly reported, 44% did not specify their techniques. 89% of JITAI designs achieved full marks on the TIDieR checklist and provided sufficient details on JITAI components. Overall, JITAIs proved to be feasible, acceptable and user‐friendly across behaviours and settings. Randomized trials showed tailored interventions were efficacious, though outcomes varied by behaviour.

**Conclusions:**

JITAIs offer a promising approach to developing personalized interventions, with their potential effects continuously growing. The recommended checklist emphasizes the importance of reporting transparency in establishing robust intervention designs.

**Statement of contribution**

**What is already known on just‐in‐time adaptive interventions?**

JITAIs approach offers guidance on developing a personalized intervention that provides the right type and dosage of support and deliver it at the right time. The potential of JITAIs is promising with studies demonstrating small benefits in physical activity and substance use. However, its efficacy and effectiveness are challenged by inconsistencies in how JITAIs are defined and characterized, as well as debates over whether existing theories effectively capture the dynamics of behaviour changes.

**What does this review add?**

- In‐depth analysis of JITAI components across various health‐related behaviours.
- Evaluation of intervention reporting quality.
- Introducing an intervention reporting checklist to improve transparency.

## INTRODUCTION

Chronic diseases such as diabetes, hypertension and cancer can negatively impact a person's quality of life (Megari, 2013), often requiring extended treatment and long‐term management. The course, severity and prognosis of chronic diseases are linked to health behaviours, including physical activity (Rhodes et al., 2017), diet (de Ridder et al., 2017), alcohol consumption (Kuntsche et al., 2017) and smoking (West, 2017). While improving health behaviours can help manage chronic diseases, behaviour change interventions often have small effect sizes and the improvement is usually temporary (Conner & Norman, 2017). This can be attributed to the complexity and individuality of health behaviours. Behaviour change is a dynamic process that requires a personalized approach, considering an individual's characteristics, needs and variability over time and in different contexts (Heino et al., 2021).

Mobile health (mHealth), which involves the use of mobile and wireless technologies like smartphones and wearables to support health care practices (World Health Organization, 2011), is increasingly being integrated into disease management (Academy of Medical Sciences (Royaume Uni), 2018). These devices can capture complex behaviour patterns and dynamic changes in real‐time over extended periods directly from patients in situ. This provides rich, individual‐specific information reflecting a person's behaviours, experiences and contexts that can be used to inform personalized support. As technology evolves, innovative methodological and intervention approaches are also emerging to facilitate personalized interventions. Ecological momentary assessment (EMA), for example, entails repeated sampling of individuals' current behaviours and experiences in their natural environements (Shiffman et al., 2008). EMA is often used alongside ecological momentary interventions (EMIs) to provide real‐time support within these everyday contexts (Heron & Smyth, 2010). While this combination enables real‐time personalized support, the specifics of how tailoring is achieved are not clear.

The concept of tailoring seeks to enhance information relevance based on individual characteristics, thereby increasing attentiveness and responsiveness to the intervention content (Dijkstra & De Vries, 1999; Kreuter & Wray, 2003). The adaptive intervention approach operationalizes the tailoring concept by introducing design and evaluation principles, including tailoring variables, decision rules and appropriate statistical methods (Collins et al., 2004). The sequential multiple assignment randomized trial (SMART) was subsequently created for developing adaptive interventions. SMART is a multistage randomized trial design that systematically evaluates different intervention sequences to establish decision rules—whether, how, when and based on what criteria to modify intervention elements such as type and dosage (e.g., duration, frequency or amount) (Almirall et al., 2014). More recently, micro‐randomized trials (MRTs) have emerged as a means to further enhance the tailoring process by optimizing the timing of intervention delivery. It involves randomly assigning various intervention options to a person at multiple time points and assessing proximal outcomes after randomization (Klasnja et al., 2015). This helps determine the optimal decision rules dictating when and under what circumstances a particular intervention option should be delivered to maximize its efficacy, while also evaluating how effects vary over time.

**FIGURE 1:**
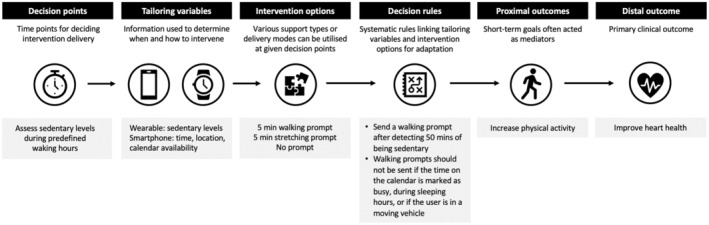
Example of a JITAI to improve heart health. It shows an example of using JITAIs to improve heart health (distal outcome) by increasing physical activity (proximal outcome). An intervention decision is made based on the user's sedentary levels, time of the day, location and calendar availability (tailoring variables), which are collected via wearable and smartphone. During waking hours (decision points), the user receives prompts (intervention options) when they have been sedentary for 50 minutes, are not in a moving vehicle and not busy (decision rules).

Building upon the crucial aspects of tailoring—content, dosage and timing—just‐in‐time adaptive interventions (JITAIs) represent an innovative approach designed to provide the right support at the right time, adapting to an individual's changing status and contexts (Nahum‐Shani et al., 2018). JITAIs are grounded in the idea that timing, identified through states of vulnerability and opportunity, plays a critical role in determining the most beneficial moments to deliver support (Nahum‐Shani et al., 2018). These moments may occur when a person is most receptive, during periods of high risk or when support is most needed. JITAIs offer a structured approach, as shown in Figure 1, with six components: (i) distal outcome (primary clinical outcome), (ii) proximal outcomes (short‐term goals often acted as mediators), (iii) decision points (time points for deciding intervention delivery), (iv) tailoring variables (information used to determine when and how to intervene), (v) intervention options (various support types or delivery modes available at given decision points) and (vi) decision rules (systematic rules linking tailoring variables and intervention options for adaptation).

The potential of JITAIs is promising, with studies demonstrating changes in physical activity (Hardeman et al., 2019) and substance use (Carpenter et al., 2020; Perski et al., 2022). However, there are challenges in defining JITAIs, adopting behaviour change theories, and establishing efficacy and effectiveness. Firstly, a wide range of interventions are classified under the JITAI umbrella. This does not necessarily imply disagreement, but rather reflects different emphasizes on varied aspects of JITAIs. For instance, Naughton defined JITAIs as context‐triggered systems that use sensors to monitor a person's context and offer support when a high‐risk situation is detected (Naughton, 2016). Adopting Naughton's definition, Hardeman and colleagues defined JITAIs as systems that deliver tailored support based on real‐time user data without relying on user initiation. The process of continually adapting support according to ongoing user data is referred to as ‘dynamic tailoring’ (Hardeman et al., 2019). Oikonomidi distinguished JITAIs by the use of algorithms to determine the timing of intervention delivery (Oikonomidi et al., 2023). The variety of JITAI definitions has highlighted important considerations of tailoring such as timing, context, real‐time adaptation and automated delivery in personalized interventions, but it can also cause discrepancies and complexities in establishing efficacy and effectiveness across JITAIs.

Secondly, while a pragmatic framework has been provided for organizing evidence in JITAIs development (Nahum‐Shani et al., 2015), the mechanisms through which JITAIs work are study‐specific. JITAIs do not strictly adhere to any theories, giving researchers the flexibility to draw on relevant evidence to inform their intervention development. This is a strength of the approach. However, adopting theories is challenging and further complicated by debates on whether existing behaviour change theories can effectively reflect the dynamic changes in an individual's behaviours, experiences and contexts (Riley et al., 2011). Finally, the efficacy and effectiveness of JITAIs are not clear. Although meta‐analyses have shown significant effect sizes favouring JITAIs over waitlist control groups and alternative treatments (Wang & Miller, 2020), systematic reviews have suggested limited evidence for their effectiveness in physical activity (Hardeman et al., 2019) and substance use (Perski et al., 2022). It is worth noting that the selection of JITAIs in existing reviews varied due to different definitions and research purposes. The heterogeneity in identification and interpretation of what constitutes JITAIs means that these reviews were assessing different interventions under the same label of JITAIs, potentially affecting the judgement of overall effects and intervention quality. The lack of clarity in intervention reporting also results in challenges in assessing intervention quality. It was reported that critical information was often missing for replication (Oikonomidi et al., 2023), and details about intervention options and the infrastructure required to implement JITAIs were insufficient for appraisal (Perski et al., 2022).

To begin addressing these challenges and facilitate the development of personalized interventions, we conducted a scoping review to identify commonalities in the development, implementation and evaluation of JITAIs for behaviour change. This review sought to build upon existing literature to achieve a more unified understanding of JITAIs and their applications in mHealth, guided by Nahum‐Shani et al. (2018), Hardeman et al. (2019) and Perski et al. (2022) teams. Our investigation specifically targeted a subset of JITAIs that identify user needs in real‐time as they occur, excluding pre‐planned opportunities. This focus was chosen because it capitalized the continuity and immediacy inherent in mHealth technologies to provide real‐time tailored support. The research questions included the following: (i) what behaviour change theories or other supporting evidence have been used to develop JITAIs? (ii) what data capturing and processing methods have been used in JITAIs? (iii) how have studies using JITAIs evaluated outcomes? Finally, we summarized the findings of randomized studies and assessed the quality of intervention reporting. Based on this, we have proposed a reporting checklist to highlight the defining characteristics of JITAIs and improve reporting transparency, aiding future research on personalized interventions.

## METHODS

The review was guided by the JBI methodology (Peters et al., 2021) and PRISMA extension for scoping reviews (Tricco et al., 2018). A search strategy was developed using the PICO model (Richardson et al., 1995) with the comparison/control category replaced by intervention methodology.

### Search strategy

A literature search of published studies was conducted in November 2021 and updated in March and December 2022 using Ovid. Relevant articles were retrieved from MEDLINE, Embase and PsycINFO. No date range was specified. We also included studies identified in the systematic reviews conducted by the Hardeman and Perski teams (Hardeman et al., 2019; Perski et al., 2022). A list of search terms was informed by previous reviews (Hardeman et al., 2019; Perski et al., 2022) and research questions. Search terms were classified into JITAIs, mHealth, behaviour change, intervention methodology and outcome evaluation (Appendix S1).

### Study selection criteria

Empirical studies of JITAIs using mHealth technologies for health behaviours in adults aged ≥18 years were included. To qualify as JITAIs, the intervention should be (i) delivered as and when a need was identified in real‐time and (ii) tailored to a person's changing needs. These included their behaviours, internal states and/or external contexts (Hardeman et al., 2019; Nahum‐Shani et al., 2018; Perski et al., 2022). We focused on real‐time needs identified in the moment as they occurred, excluding pre‐planned opportunities. The selection criteria ensured that the intervention was immediately relevant and continually adapted to the individual's current circumstances. No restrictions for intervention triggers (e.g., user‐triggered, server‐triggered or hybrid), monitoring methods (e.g., active or passive) and monitoring frequency (e.g., at random times or fixed intervals), provided that the support matched user needs. Both stand‐alone and adjunct interventions were included. Reviews, commentaries, editorials, dissertations, protocols, book chapters, conference abstracts, case studies, secondary analyses, conceptual and methodological articles, and non‐English articles were excluded.

### Screening, data charting and synthesis

**FIGURE 2:**
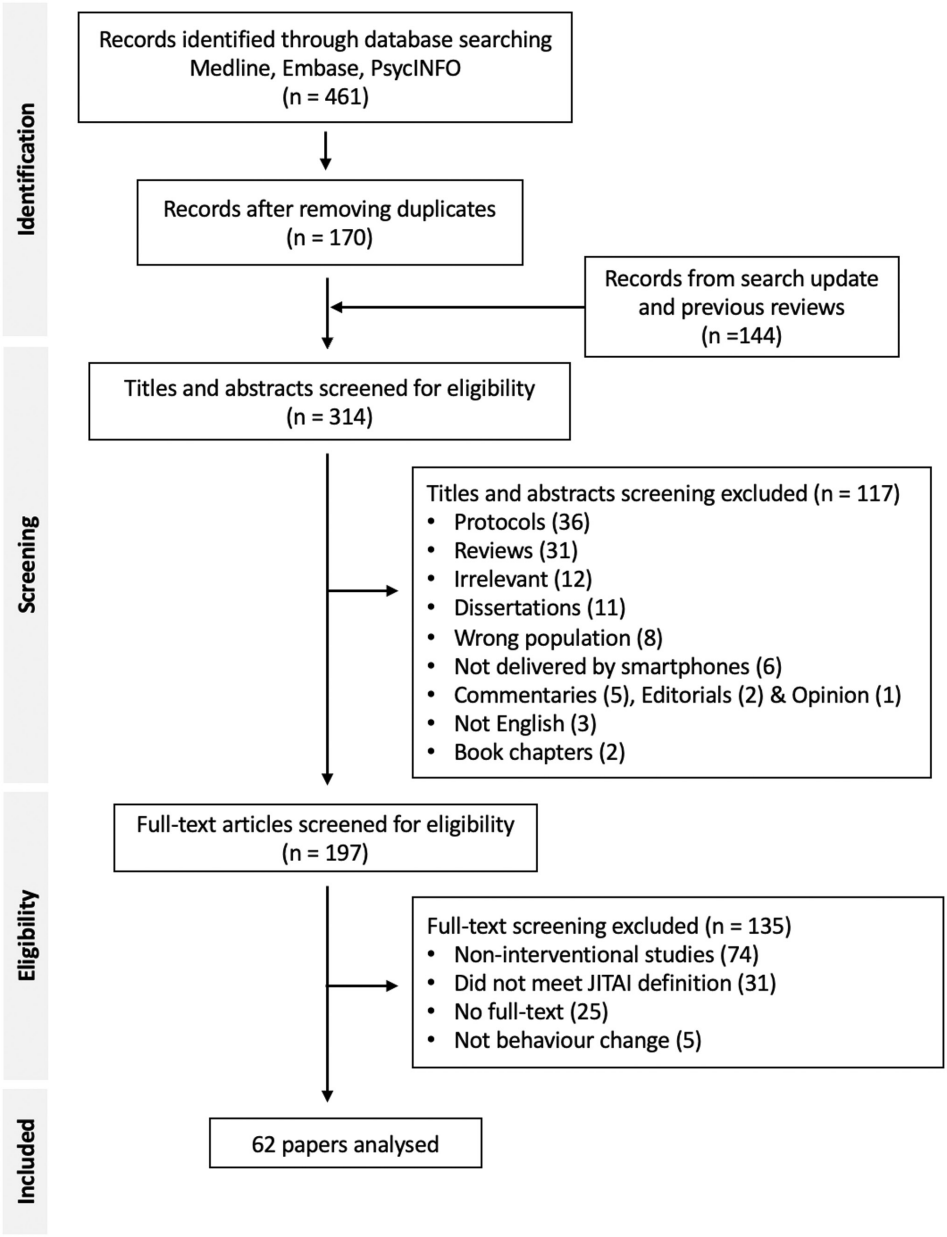
Flow diagram of study selection. It illustrates the process of study selection using two criteria: the intervention should be (i) delivered as and when a need was identified in real‐time and (ii) tailored to a person's changing needs. These needs included their behaviours, internal states and/or external contexts.

All identified articles including the subsequent updates were collated and screened using Rayyan (Ouzzani et al., 2016). After removing duplicates, the first author conducted titles and abstracts screening, followed by full‐text screening for eligibility. The full‐text screening was double‐reviewed by co‐author JG, who randomly assessed 20% of articles against the inclusion criteria (Agreement rate = 89.3%, κ = 0.78). Disagreements were resolved through team discussion. Figure 2 shows the selection process.

A data‐charting form was jointly developed by the research team. Data items were selected based on research questions (Appendix S2). Study details included article information (first author, year of publication and location), research purpose, research design, participant characteristics (age and sex), intervention type (how authors described their interventions, e.g., JITAIs, EMIs), intervention app, intervention duration, target condition and target behaviours. JITAI components included decision points, tailoring variables, decision rules, intervention options, proximal outcomes and distal outcomes. Key elements from research questions included supporting evidence (evidence cited for JITAI development), evidence‐informed JITAI components (JITAI components informed by the cited evidence), data capturing methods (how user information is measured in real‐time for tailoring variables, e.g., self‐reporting, passive monitoring or a hybrid approach), data processing methods (how user information is processed in real‐time for decision rules), evaluation methods of feasibility, acceptability and usability, and intervention/control groups (for randomized studies). A narrative synthesis was conducted given the heterogeneity of research purposes, designs, behaviours, target populations and outcomes.

### Quality of intervention reporting

We used the template for intervention description and replication (TIDieR) checklist (Hoffmann et al., 2014), which is a 12‐item checklist developed as an extension of the CONSORT 2010 statement (item 5) (Schulz et al., 2010) and the SPIRIT 2013 statement (item 11) (Chan et al., 2013) for improving the completeness of reporting. The checklist criteria included intervention name, rationale of intervention, intervention materials and procedures, intervention provider, delivery mode and setting, intervention details (e.g., duration, frequency, intensity, dose), whether the intervention was tailored or modified, and methods to assess adherence or fidelity (Hoffmann et al., 2014). The assessment was conducted by the first author, with co‐author JMcB randomly evaluating 20% of JITAI designs (Agreement rate = 94.8%). Disagreements were resolved through discussion.

For the tailoring (item 9) in the TIDieR checklist, we provided additional assessment regarding the JITAI components, namely decision points, tailoring variables, intervention options, decision rules, proximal outcomes and distal outcome (Nahum‐Shani et al., 2018). The assessment focused on the clarity of intervention components (whether each JITAI component was sufficiently described). A JITAIs reporting checklist was created by adapting the TIDieR checklist and incorporating key findings from our analysis. Appendices S7 and S8 contain the full assessment records.

## RESULTS

A total of 461 studies were identified from Medline, Embase and PsycINFO in November 2021. After removing duplicates, 170 studies were available for screening. The search was repeated twice in March and December 2022, including in press and online first publications. Studies identified in systematic reviews were also included. This resulted in 144 additional studies. Overall, 314 publications were screened by titles and abstracts for relevance, 117 (37%) were excluded, leaving 197 for full‐text screening. The primary reasons for exclusion at this stage were protocols, review articles or topics irrelevant to personalized interventions. Of those, 62 studies published between 2011 and 2023 were eligible and included in the synthesis. The majority of studies that were excluded were non‐interventional studies, did not meet our selection criteria, no full‐text, or did not include behaviour change (Figure 2).

### Study characteristics

Of the 62 studies classified as JITAIs (Table 1), the reported intervention types included EMIs (*n* = 21), JITAIs (*n* = 18), mHealth interventions (*n* = 18), text‐messaging interventions (*n* = 3), just‐in‐time intervention (*n* = 1) and intelligent real‐time therapy (*n* = 1). The majority of studies were conducted in the United States (48/62, 77%), nine in Europe (UK, Netherlands, France), two in Uganda and Australia, respectively, and one in Qatar. There were 44 studies (71%) designed to evaluate feasibility, acceptability and/or usability, 16 (26%) designed to assess intervention efficacy (*n* = 11) and effectiveness (*n* = 5), and one to investigate engagement and intervention delivery timing, respectively. Target behaviours included substance use (e.g., tobacco, alcohol, drugs) (18/62, 29%), self‐management skills (8/62, 12.9%), physical activity (7/62, 11.3%), dietary behaviour (7/62, 11.3%), sedentary behaviour (6/62, 9.7%), treatment adherence (4/62, 6.5%), sexual behaviour (2/62, 3.2%), fluid consumption (1/62, 1.6%), social skills (1/62, 1.6%), gambling behaviour (1/62, 1.6%) and multiply targeted lifestyle behaviours (JITAIs targeting multiple health‐related behaviours; 7/62, 11.3%). The average mean age across studies was 37.3 years (range: 19.2–55.3), and on average, 59.9% of participants were female (range: 17–100%). The average sample size was 2033 (range: 3–119,713). The average study duration was 69.4 days (range: 1 week to 13 months).

**TABLE 1:** Characteristics of included studies.

First author, year, country	Intervention type (app)	Research purposes	Research design	Target conditions	Target behaviours	Intervention duration	Participant's characteristics
Wang et al. (2023), US	mHealth intervention (Intern+)	Effectiveness	MRT	None	Mood, physical activity, sleep	12 weeks	1779 adults, 54.5% female, mean age 27.6
Yang et al. (2023), US	JITAI (mCerebrum)	Feasibility Acceptability	MRT	Daily smokers	Smoking cessation	6 weeks (JITAIs: first 2 weeks)	43 adults, 58.1% female, mean age 49.1
Beres et al. (2022), US/Uganda	EMAI	Feasibility Acceptability	Pilot RCT	None	Fruit and vegetable consumption, drinking, smoking and sexual behaviour	90 days (JITAIs: start at day 30)	48 adults, 48% female, median age 31
Carlozzi et al. (2022), US	JITAI (CareQOL)	Feasibility Acceptability	RCT	None	Physical activity, sleep hygiene, mood	90 days	70 care partners, 70% female, mean age 55.3
Ismail & Al (2022), Qatar	JITAI (MotiFit)	Effectiveness	Mixed‐methods between group design	None	Sedentary behaviour	66 days	58 adults, 62% female, aged 23–30
Juarascio et al. (2022), US	JITAI (SenseSupport)	Feasibility Acceptability	ABAB design	Binge eating disorder and bulimia nervosa	Dietary behaviour	12 weeks	30 adults, 87% female, mean age 37.1
Mair et al. (2022), UK	JITAI (JitaBug)	Feasibility Acceptability	Mixed‐methods pretest‐to‐posttest trial	None	Physical activity	6 weeks (JITAIs: 4 weeks)	31 adults, 55% female, aged 56–72
Morgiève et al. (2022), France	EMA/EMI (EMMA)	Acceptability Satisfaction	Prospective, longitudinal and multicentric study	Suicide ideation or attempt	Suicidal behaviour	6 months	75 adults, 76% female, mean age 32.4
Sizemore et al. (2022), US	JITAI EMI (LifeData RealLifeExp)	Feasibility Acceptability Usability	Proof‐of‐concept pilot MRT	HIV	Self‐management skills	90 days	22 men, mean age 37.8
Walters et al. (2022), US	JITAI (Smart‐T Alcohol)	Feasibility	Single‐arm pilot trial	Homelessness	Alcohol use	4 weeks	41 adults, 19.5% female, mean age 45.2
Beres et al. (2021), US/Uganda	EMAI	Feasibility Estimate effect	Pilot RCT	None	Fruit and vegetable consumption, drinking, smoking and sexual behaviour	90 days (JITAIs: start at day 30)	48 adults, 48% female, median age 31
Blevins et al. (2021), US	EMI (mEMA)	Feasibility Acceptability	Mixed‐methods feasibility study	Alcohol use	Alcohol use	6 weeks	20 emerging adults, 55% female, mean age 21.7
Coughlin et al. (2021), US	JITAI (MiSARA)	Feasibility Acceptability	Pre–post within group design	Binge drinking or recreational cannabis use	Substance use	1 month	39 young adults, 62% female, mean age 20.7
Fulford et al. (2021), US	EMI (MASS)	Preliminary efficacy	Open pilot trial	Schizophrenia	Social skills	8 weeks	31 adults, 48% female, mean age 46
Gire et al. (2021), UK	iRTT (TechCare)	Feasibility Acceptability	Mixed‐methods feasibility pilot trial	Psychosis	Self‐management skills	6 weeks	12 adults (with 4 test‐run), 33.3% female, mean age 24.8
Hawker et al. (2021), Australia	EMI (MetricWire)	Feasibility and acceptability	Single‐arm trial	Gambling problem	Gambling behaviour	5 weeks	36 adults, 39% female, age 18+ (47% between 35 and 49)
Juarascio, Srivastava, et al. (2021), US	JITAI (CBT+)	Feasibility Acceptability Preliminary clinical outcomes	Clinician‐controlled pilot trial	Bulimia nervosa	Dietary behaviour	16 weeks	3 adults; 2 clinicians, 80% female, mean age 35.6
Juarascio, Hunt, et al. (2021), US	EMI (iCAT+)	Feasibility Acceptability	Mixed‐methods open pilot trial	Binge eating disorder and bulimia nervosa	Dietary behaviour	Approx. 19 weeks (21 sessions)	16 female adults, mean age 34.1
Santa Maria et al. (2021), US	JITAI (MY‐RID)	Efficacy	Pilot RCT	Homelessness	HIV risk behaviour	6 weeks	97 young adults, 35.1% female, mean age 21.2
Wang et al. (2021), The Netherlands	JITAI (PAUL)	Feasibility	Mixed‐methods feasibility study	None	Physical activity	4 weeks (JITAIs: 1 week)	7 adults, 71.4% female, mean age 34.4
Conroy et al. (2020), US	JITAI (sipIT)	Acceptability	Single‐group trial	Kidney stones	Fluid consumption	3 months	31 adults, 58% female, mean age 40
Hébert et al. (2020), US	JITAI (Smart‐T2)	Feasibility Preliminary efficacy	Pilot RCT	Smokers	Smoking cessation	13 weeks	81 adults, 51% female, mean age 49.6
Low et al. (2020), US	Real‐time mobile intervention (DASH)	Acceptability Usability	Single‐arm pilot trial	Abdominal cancer surgery	Sedentary behaviour	At least 2 weeks	15 adults, 80% female, mean age 49.7
NeCamp et al. (2020), US	mHealth intervention	Intervention delivery timing	MRT	None	Mood, physical activity, sleep	26 weeks	1565 medical interns, 56% female
Scott et al. (2020), US	EMI (Adapted A‐CHESS)	Effectiveness	RCT	Substance use disorders	Risk behaviour for substance use	6 months	401 adults, 39% female, mean age 44
Shrier et al. (2020), US	EMI (MARSSI)	Feasibility Acceptability	Mixed‐methods pilot study	Depressive symptoms	High‐risk sexual behaviour	4 weeks	17 young women, median age 20.6
Valle et al. (2020), US	mHealth intervention (The Nudge)	Characterizing engagement	MRT	Overweight and sedentary lifestyle	Dietary behaviour	12 weeks	53 young adults, 79.2% female, mean age 29.6
Bartlett Ellis et al. (2019), US	mHealth system (Pebblebee)	Feasibility and acceptability	Single‐arm trial	Chronic kidney disease	Treatment adherence	52 days	5 adults, 40% female, mean age 52.6
Forman, Goldstein, Zhang, et al. (2019), US	JITAI (Weight Watcher + OnTrack)	Feasibility Acceptability Preliminary effectiveness	Open trial	Overweight and obesity	Dietary behaviour	8 weeks	43 adults, 86% female, mean age 51
Forman, Goldstein, Crochiere, et al. (2019), US	JITAI (Weight Watcher + OnTrack)	Efficacy	RCT	Overweight and obesity	Dietary behaviour	10 weeks	181 adults, 85.1% female, mean age 46.3
Hiremath et al. (2019), US	JITAI (PHIRE)	Feasibility and acceptability	Single‐arm trial	Spinal cord injury	Physical activity	1 month	20 adults, 20% female, mean age 39.4
Hoeppner et al. (2019), US	Text‐messaging intervention (SmokefreeTXT)	Acceptability and user experience	Single‐arm trial	Non‐daily smokers	Smoking cessation	6 weeks	32 adults, 64% female, mean age 35
Klasnja et al. (2019), US	JITAI MRT (HeartSteps)	Efficacy	MRT	Sedentary lifestyle	Physical activity	6 weeks	44 adults (37 analysed), 70.5% female, mean age 35.9
Levin, Haeger, & Cruz (2019), US	EMA (ACT Daily)	Efficacy	RCT	None	Self‐management skills	4 weeks	69 adults, 68.1% female, mean age 21.9
Levin, Navarro, et al. (2019), US	EMA (ACT Daily)	In‐the‐moment effects	RCT	None	Self‐management skills	4 weeks	39 adults, 60% female, mean age 21.9
O'Donnell et al. (2019), Australia	mHealth intervention (Minimize)	Feasibility and effects	Single‐blind RCT	Alcohol use	Alcohol use	28 days	45 young adults, 80% female, mean age 22.1
Hébert et al. (2018), US	EMI (Smart‐T)	Utility	Single‐arm trial	Smokers	Smoking cessation	3 weeks	59 adults, 54.2% female, mean age 52
Shrier et al. (2018), US	EMI (MOMENT)	Feasibility, acceptability and preliminary efficacy	Pilot parallel‐group trial with randomization	Marijuana use	Marijuana use	2 weeks	70 young adults, 60% female, mean age 20.7
Van Dantzig et al. (2018), The Netherlands	mHealth intervention	Effectiveness and user experience	RCT	None	Physical activity	4 weeks (JITAIs: 1 week)	70 adults, 27% female, age between 18 and 65
Attwood et al. (2017), UK	mHealth intervention (Drinkaware)	Quant: engagement and user pattern Qual: acceptability, usability and perceived effectiveness	Mixed‐methods sequential explanatory design	Alcohol use	Alcohol use	13 months	119,713 adults, 59.3% female, age 18+ (31% between 35 and 44)
Leonard et al. (2017), US	EMI (MtM)	Feasibility Acceptability	Single‐arm pilot study	None	Alcohol use	3–4 weeks	10 female undergraduate, mean age 20.7
Businelle et al. (2016), US	EMI (Smart‐T)	Feasibility Preliminary effectiveness	Nonrandomized feasibility study	Smokers	Smoking cessation	3 weeks (1 week not tailored)	59 adults, 54% female, mean age 52
Ding et al. (2016), US	JIT intervention (WalkMore)	Acceptability, usability and effectiveness	Pilot RCT	None	Physical activity	4 weeks (JITAIs: 3 weeks)	16 young adults, 37.5% female, age between 18 and 25
Naughton et al. (2016), UK	Context‐aware EMI (Q Sense)	Feasibility	Explanatory sequential mixed‐methods design	Smokers	Smoking cessation	At least 34 days (JITAIs: after post‐quit day, lasting 28 days)	15 adults, 47% female, aged 18 and over (60% aged 25–34)
Wenze et al. (2016), US	EMI (MyT)	Feasibility Acceptability	Mixed‐methods open trial	Bipolar disorder	Treatment adherence	2 months	8 adults, 62.5% female, mean age 44
Depp et al. (2015), US	EMI	Feasibility, acceptability and efficacy	Single‐blind RCT	Bipolar disorder	Self‐management skills	10 weeks	82 adults, 58.5% female, mean age 47.5
Finkelstein et al. (2015), US	mHealth intervention	Potential efficacy	Randomized crossover design	Overweight and sedentary	Sedentary behaviour	8 weeks	27 adults, 100% female, mean age 52
Gonzalez & Dulin (2015), US	mHealth intervention (LBMI‐A)	Short‐term effects	Single‐arm sequential pilot study	Alcohol use disorder	Alcohol use	6 weeks	48 adults, 41.7% female, mean age 33.9
Ingersoll et al. (2015), US	Text‐messaging intervention (TxText)	Preliminary efficacy	Pilot RCT	HIV	Treatment adherence	12 weeks	63 adults, 39.7% female, mean 42.4
Mundi et al. (2015), US	EMI	Feasibility	Feasibility trial	Bariatric surgery	Dietary behaviour	12 weeks	30 adults (20 completed), 90% female, mean age 41.3
Pellegrini et al. (2015), US	mHealth intervention (NEAT!)	Feasibility and acceptability	Single‐arm pilot trial	Type 2 Diabetes	Sedentary behaviour	1 month	9 adults, 77% female, mean age 53.1
Rabbi, Pfammatter, et al. (2015), US	mHealth intervention (MyBehavior)	Feasibility and usability	RCT	None	Physical activity and dietary behaviour	3 weeks	17 adults, 47% female, mean age 28.3
Rabbi, Aung, et al. (2015), US	mHealth intervention (MyBehavior 2.0)	Efficacy	Multiple baseline design	None	Physical activity and dietary behaviour	14 weeks (JITAIs: week 7–9)	16 adults, 56.3% female, age 18+
Thomas & Bond (2015), US	JITAI (B‐MOBILE)	Intervention effects	Randomized counterbalanced design	Overweight and obesity	Sedentary behaviour	21 days	30 adults, 83% female, mean age 47.5
Ben‐Zeev et al. (2014), US	mHealth intervention (FOCUS)	Feasibility, acceptability and preliminary efficacy	Single‐arm trial	Schizophrenia	Self‐management skills	1 month	33 adults, 39% female, mean age 45.9
Dulin et al. (2014), US	mHealth intervention (LBMI‐A)	Early‐stage effectiveness and usability	Single‐arm pilot trial	Alcohol use disorder	Alcohol use	6 weeks	28 adults, 46.4% female, mean age 33.6
Gustafson et al. (2014), US	mHealth intervention (A‐CHESS)	Effectiveness	Unblinded RCT	Alcohol use disorder	Alcohol use	8 months	349 adults, 39% female, mean age 38
Ingersoll et al. (2014), US	Text‐messaging intervention (TxText)	Feasibility and acceptability	Pilot RCT, usability trial	HIV	Treatment adherence	10 months	57 adults, 40.4% female, mean age 42.1
Shrier et al. (2014), US	EMI (MOMENT)	Feasibility, acceptability and potential efficacy	Single‐arm pilot trial	Marijuana use	Marijuana use	2 weeks	27 young adults, 70% female, mean age 19.2
Van Dantzig et al. (2013), The Netherlands	mHealth intervention (SitCoach)	Usability and effects	RCT	None	Sedentary behaviour	6 weeks	86 adults, 39.5% female, mean age 44.4
Burns et al. (2011), US	EMI (Mobilyze!)	Feasibility Reliability Satisfaction	Single‐arm field trial	Major depression disorder	Self‐management skills	8 weeks	8 adults, 87.5% female, mean age 37.4
Lin et al. (2011), The Netherlands	mHealth system (Motivate)	Feasibility and usability	Single‐arm trial	None	Physical activity	5 weeks	6 adults, 17% female, mean age 37

### Supporting evidence for behaviour change

In total, we identified relevant evidence supporting JITAIs development in 46 studies (74%), many of which drew from behaviour sciences (Appendix S3). Mostly commonly, behaviour change techniques were identified in seven studies across dietary behaviour (Forman, Goldstein, Crochiere, et al., 2019; Forman, Goldstein, Zhang, et al., 2019; Valle et al., 2020), smoking cessation (Hoeppner et al., 2019; Naughton et al., 2016), alcohol use (Attwood et al., 2017), and physical activity and sleep (Wang et al., 2023). Other theoretical perspectives included Fogg behaviour model (*n* = 4) (Ding et al., 2016; NeCamp et al., 2020; Rabbi, Aung, et al., 2015; Rabbi, Pfammatter, et al., 2015), habit formation (*n* = 3) (Bartlett Ellis et al., 2019; Ding et al., 2016; Ismail & Al, 2022), information, motivation and behaviour skills model (*n* = 3) (Ingersoll et al., 2014, 2015; Santa Maria et al., 2021), learning theory (*n* = 3) (Naughton et al., 2016; Rabbi, Aung, et al., 2015; Rabbi, Pfammatter, et al., 2015), operant conditioning (*n* = 3) (Conroy et al., 2020; Coughlin et al., 2021; Hiremath et al., 2019), behavioural activation approach (*n* = 2) (Burns et al., 2011; Carlozzi et al., 2022), behaviour change wheel (*n* = 2) (Mair et al., 2022; Wang et al., 2021), self‐determination theory (*n* = 2) (Gustafson et al., 2014; Scott et al., 2020), social cognitive theory (*n* = 2) (Rabbi, Aung, et al., 2015; Rabbi, Pfammatter, et al., 2015), social action theory (*n* = 2) (Ingersoll et al., 2014, 2015) and nudging (*n* = 2) (Beres et al., 2021, 2022).

Established treatments were also identified. For example, cognitive behavioural therapy was used in eight studies across dietary behaviour (Juarascio et al., 2022; Juarascio, Srivastava, et al., 2021), alcohol use (Blevins et al., 2021; Leonard et al., 2017), sexual behaviour (Shrier et al., 2020), gambling behaviour (Hawker et al., 2021), social skills (Fulford et al., 2021) and self‐management skills (Gire et al., 2021). Other therapeutical approaches included motivational interviewing (*n* = 6) (Dulin et al., 2014; Gonzalez & Dulin, 2015; Hawker et al., 2021; Leonard et al., 2017; NeCamp et al., 2020; Shrier et al., 2020), acceptance and commitment therapy (*n* = 2) (Levin, Haeger, & Cruz, 2019; Levin, Navarro, et al., 2019), motivational enhancement therapy (*n* = 2) (Shrier et al., 2014, 2018), integrative cognitive‐affective therapy (*n* = 1) (Juarascio, Hunt, et al., 2021) and protective behavioural strategies (*n* = 1) (O'Donnell et al., 2019). Among the identified evidence, 31 studies (67%) reported applying that evidence exclusively to developing intervention options. Other evidence‐informed aspects included tailoring variables, decision rules, proximal outcomes, intervention apps, engagement strategies and goal identification.

### Real‐time data capturing and processing

To identify user needs in real‐time, JITAIs gather individual information as tailoring variables through three methods: self‐reporting, passive monitoring or a combination of both called the hybrid approach (Appendix S4). We found that 34 JITAIs (55%) relied on self‐reporting only, with 65% (22/34) reporting using EMA or experiential sampling method, a technique akin to EMA that captures subjective experiences as they occur in everyday life (Csikszentmihalyi et al., 2014). Of the remaining studies, 15 (24%) adopted a hybrid approach and 13 (21%) used only passive monitoring. Studies that exclusively employed passive monitoring included physical activity (*n* = 6) (Ding et al., 2016; Klasnja et al., 2019; Lin et al., 2011; Mair et al., 2022; Van Dantzig et al., 2018; Wang et al., 2021), sedentary behaviour (*n* = 4) (Finkelstein et al., 2015; Ismail & Al, 2022; Thomas & Bond, 2015; Van Dantzig et al., 2013), substance use (*n* = 2) (Gustafson et al., 2014; Yang et al., 2023) and fluid consumption (*n* = 1) (Conroy et al., 2020).

Self‐initiated reporting was common across studies (23%, 14/62), allowing users to trigger support as needed. Two studies required participants to actively report their daily dietary behaviour (Juarascio, Srivastava, et al., 2021) and medications (Bartlett Ellis et al., 2019) to trigger JITAIs. All JITAIs that collected objective data utilized non‐invasive mHealth devices in addition to smartphones. Activity tracker was the mostly commonly used (Carlozzi et al., 2022; Conroy et al., 2020; Ding et al., 2016; Finkelstein et al., 2015; Hiremath et al., 2019; Klasnja et al., 2019; Low et al., 2020; Mair et al., 2022; NeCamp et al., 2020; Pellegrini et al., 2015; Valle et al., 2020; Van Dantzig et al., 2013, 2018; Wang et al., 2023); other devices included continuous glucose monitoring device (Juarascio et al., 2022), electrodermal activity tracker (Leonard et al., 2017), wireless weight scale (Valle et al., 2020), sensors detecting wrist movement, electrocardiography and respiration (Yang et al., 2023), and connected water bottle (Conroy et al., 2020). Although these data capturing techniques primarily served to measure tailoring variables, some techniques also overlapped with those used in evaluating proximal outcomes.

Several techniques were identified for JITAIs to process real‐time information and make adaptive decisions. Over half of the included studies (56%, 35/62) provided the purpose, development or source of chosen processing techniques. Seven studies (17%) reported using machine learning algorithms for identifying real‐time needs and timing to deliver tailored support. These JITAIs targeted physical activity (Hiremath et al., 2019; Wang et al., 2021), alcohol use (Walters et al., 2022), dietary behaviour (Forman, Goldstein, Crochiere, et al., 2019; Forman, Goldstein, Zhang, et al., 2019) and self‐management skills (Burns et al., 2011; Gire et al., 2021).

### Outcome evaluations

The majority of JITAIs (71%) were conducted to evaluate feasibility, acceptability and/or usability, with a subset (*n* = 9) also reporting preliminary effects (Ben‐Zeev et al., 2014; Beres et al., 2021; Businelle et al., 2016; Forman, Goldstein, Zhang, et al., 2019; Hébert et al., 2020; Juarascio, Srivastava, et al., 2021; Shrier et al., 2014, 2018; Walters et al., 2022). Feasibility was commonly evaluated by objective measurements such as compliance, adherence, retention, app usage or intervention usage. Acceptability and usability were assessed by both subjective and objective measurements. Subjective responses for acceptability included perception, satisfaction, helpfulness, usefulness, ease of use or likeability, whereas objective responses included response rate and app usage. Usability was measured through objective behaviour and engagement metrics such as app usage, participation in the intervention, response rate and completion rate, while subjective responses included helpfulness, usefulness, satisfaction, easiness and pleasantness (Appendix S5).

Standardized questionnaires were identified in 15 studies (24%) to quantify acceptability and usability. For assessing acceptability, the instruments included the client satisfaction questionnaire (*n* = 5) (Hawker et al., 2021; Leonard et al., 2017; Sizemore et al., 2022; Wenze et al., 2016; Yang et al., 2023), technology acceptance model scales (*n* = 4) (Ben‐Zeev et al., 2014; Forman, Goldstein, Crochiere, et al., 2019; Forman, Goldstein, Zhang, et al., 2019; Juarascio, Hunt, et al., 2021), mobile application rating scale (*n* = 2) (Hawker et al., 2021; Morgiève et al., 2022) and user burden scale (*n* = 1) (Conroy et al., 2020). Usability assessments included the system usability scale (*n* = 7) (Ben‐Zeev et al., 2014; Conroy et al., 2020; Ding et al., 2016; Levin, Haeger, & Cruz, 2019; O'Donnell et al., 2019; Sizemore et al., 2022; Yang et al., 2023) and mobile application rating scale (*n* = 1) (Ismail & Al, 2022). Due to the heterogeneity in metrics and assessment frequencies, direct comparisons of outcomes across studies were not conducted. However, there was a consensus that JITAIs were feasible, acceptable and user‐friendly.

### Potential effects of JITAIs

Of the nine studies reporting preliminary effects alongside feasibility and acceptability, two employed pilot RCTs (Beres et al., 2021; Hébert et al., 2020). The findings showed that JITAIs appeared to have a positive effect, though results varied across behaviours. For example, Beres's study (Beres et al., 2021), which targeted smoking, risky sexual behaviour, and fruit, vegetable and alcohol consumption, reported decreases in alcohol intake and risky sexual behaviour in both groups over time. However, increased vegetable consumption during the same period was observed only in the tailored message group. This group also showed a significantly greater increase in vegetable consumption compared to the self‐tracking group, while no significant changes were noted in the remaining behaviours. In contrast, Hébert's study (Hébert et al., 2020) on smoking cessation found no significant differences in smoking abstinence across tailored, non‐tailored and usual care groups.

A total of 16 studies (20%) investigated effectiveness and efficacy of JITAIs in substance use (Gonzalez & Dulin, 2015; Gustafson et al., 2014; Scott et al., 2020), physical activity (Klasnja et al., 2019; Rabbi, Aung, et al., 2015; Van Dantzig et al., 2018; Wang et al., 2023), sedentary behaviour (Finkelstein et al., 2015; Ismail & Al, 2022; Thomas & Bond, 2015), sexual behaviour (Santa Maria et al., 2021), dietary behaviour (Forman, Goldstein, Crochiere, et al., 2019; Rabbi, Aung, et al., 2015), treatment adherence (Ingersoll et al., 2015), social skills (Fulford et al., 2021) and self‐management skills (Levin, Haeger, & Cruz, 2019; Levin, Navarro, et al., 2019). Out of these, there were five RCTs (Forman, Goldstein, Crochiere, et al., 2019; Gustafson et al., 2014; Levin, Haeger, & Cruz, 2019; Levin, Navarro, et al., 2019; Van Dantzig et al., 2018) and two pilot RCTs (Ingersoll et al., 2015; Santa Maria et al., 2021). The overall findings suggested that JITAIs were more effective than their comparison groups. For instance, personalized messages demonstrated greater treatment adherence compared to usual care (Ingersoll et al., 2015), as well as significant reductions in drug use and stress, and a lower urge for risky sexual behaviour, when compared to random messages (Santa Maria et al., 2021). Similar findings were reported with significant reductions in risky drinking days when real‐time alerts for risky locations were used alongside usual treatment, as opposed to usual treatment alone (Gustafson et al., 2014). Also, tailored skill coaching using ACT led to significant improvements in psychological distress and functioning relative to non‐tailored coaching (Levin, Haeger, & Cruz, 2019; Levin, Navarro, et al., 2019), and a tailored risk prevention programme achieved greater weight loss than a standard digital programme (Forman, Goldstein, Crochiere, et al., 2019). Despite these successes, a tailored coaching system designed for physical activity observed an increase in average daily steps, but this increase was not significantly different from that of the general advice group (Van Dantzig et al., 2018).

Regarding proximal effects, two MRTs were conducted on physical activity and sleep (Klasnja et al., 2019; Wang et al., 2023). Findings of gamification showed that, under the same personalized feedback mechanism, the competition teams significantly increased their daily step counts compared to teams without competition. However, this did not affect daily sleep duration. Additionally, providing activity suggestions was found to increase average step counts, though the effect diminished over time, while anti‐sedentary suggestions showed no detectable effect (Klasnja et al., 2019). Appendix S6 details the intervention and control groups.

### Quality of intervention reporting

From the included studies, 55 unique JITAI designs were identified. We found all JITAI designs reported on all 12 items in the TIDieR checklist. Further assessment on the clarity of intervention components showed that the majority (89%, 49/55) provided sufficient details on all JITAI components. However, we were unable to identify clear details in six JITAI designs in terms of their tailoring variables (*n* = 1), intervention options (*n* = 3) and decision rules (*n* = 3). Overall, 89% achieved full marks on the TIDieR checklist and provided adequate information on JITAI components (Table 2).

**TABLE 2:** Quality of intervention reporting.

Included studies	Target conditions	Target behaviours	TIDieR	Clarity
Wang et al. (2023)	None	Mood, physical activity, sleep	100%	100%
Yang et al. (2023)	Daily smokers	Smoking cessation	100%	100%
Beres et al. (2021, 2022)	None	Fruit and vegetable consumption, drinking, smoking and sexual behaviour	100%	83%
Carlozzi et al. (2022)	None	Physical activity, sleep hygiene, mood	100%	100%
Ismail & Al (2022)	None	Sedentary behaviour	100%	100%
Juarascio et al. (2022)	Binge eating disorder and bulimia nervosa	Dietary behaviour	100%	83%
Mair et al. (2022)	None	Physical activity	100%	100%
Morgiève et al. (2022)	Suicide ideation or attempt	Suicidal behaviour	100%	100%
Sizemore et al. (2022)	HIV	Self‐management skills	100%	100%
Walters et al. (2022)	Homelessness	Alcohol use	100%	100%
Blevins et al. (2021)	Alcohol use	Alcohol use	100%	100%
Coughlin et al. (2021)	Binge drinking or recreational cannabis use	Substance use	100%	100%
Fulford et al. (2021)	Schizophrenia	Social skills	100%	100%
Gire et al. (2021)	Psychosis	Self‐management skills	100%	83%
Hawker et al. (2021)	Gambling problem	Gambling behaviour	100%	100%
Juarascio, Srivastava, et al. (2021)	Bulimia Nervosa	Dietary behaviour	100%	100%
Juarascio, Hunt, et al. (2021)	Binge eating disorder and bulimia nervosa	Dietary behaviour	100%	100%
Santa Maria et al. (2021)	Homelessness	HIV risk behaviours	100%	100%
Wang et al. (2021)	None	Physical activity	100%	100%
Conroy et al. (2020)	Kidney stones	Fluid consumption	100%	100%
Hébert et al. (2020)	Smokers	Smoking cessation	100%	100%
Low et al. (2020)	Abdominal cancer surgery	Sedentary behaviour	100%	100%
NeCamp et al. (2020)	None	Mood, physical activity, sleep	100%	100%
Scott et al. (2020)	Substance use disorders	Risk behaviour for substance use	100%	100%
Shrier et al. (2020)	Depressive symptoms	High‐risk sexual behaviour	100%	100%
Valle et al. (2020)	Overweight and sedentary lifestyle	Dietary behaviour	100%	100%
Bartlett Ellis et al. (2019)	Chronic kidney disease	Treatment adherence	100%	100%
Forman, Goldstein, Crochiere, et al. (2019), Forman, Goldstein, Zhang, et al. (2019)	Overweight and obesity	Dietary behaviour	100%	100%
Hiremath et al. (2019)	Spinal cord injury	Physical activity	100%	100%
Hoeppner et al. (2019)	Non‐daily smokers	Smoking cessation	100%	67%
Klasnja et al. (2019)	Sedentary lifestyle	Physical activity	100%	100%
Levin, Haeger, & Cruz (2019), Levin, Navarro, et al. (2019)	None	Self‐management skills	100%	100%
O'Donnell et al. (2019)	Alcohol use	Alcohol use	100%	100%
Hébert et al. (2018)	Smokers	Smoking cessation	100%	100%
Shrier et al. (2014, 2018)	Marijuana use	Marijuana use	100%	100%
Van Dantzig et al. (2018)	None	Physical activity	100%	100%
Attwood et al. (2017)	Alcohol use	Alcohol use	100%	100%
Leonard et al. (2017)	None	Alcohol use	100%	100%
Businelle et al. (2016)	Smokers	Smoking cessation	100%	100%
Ding et al. (2016)	None	Physical activity	100%	100%
Naughton et al. (2016)	Smokers	Smoking cessation	100%	100%
Wenze et al. (2016)	Bipolar disorder	Treatment adherence	100%	100%
Depp et al. (2015)	Bipolar disorder	Self‐management skills	100%	100%
Finkelstein et al. (2015)	Overweight and sedentary	Sedentary behaviour	100%	100%
Gonzalez & Dulin (2015), Dulin et al. (2014)	Alcohol use disorder	Alcohol use	100%	100%
Ingersoll et al. (2014, 2015)	HIV	Treatment adherence	100%	100%
Mundi et al. (2015)	Bariatric surgery	Dietary behaviour	100%	83%
Pellegrini et al. (2015)	Type 2 Diabetes	Sedentary behaviour	100%	100%
Rabbi, Aung, et al. (2015), Rabbi, Pfammatter, et al. (2015)	None	Physical activity and dietary behaviour	100%	100%
Thomas & Bond (2015)	Overweight and obesity	Sedentary behaviour	100%	100%
Ben‐Zeev et al. (2014)	Schizophrenia	Self‐management skills	100%	83%
Gustafson et al. (2014)	Alcohol use disorder	Alcohol use	100%	100%
Van Dantzig et al. (2013)	None	Sedentary behaviour	100%	100%
Burns et al. (2011)	Major depression disorder	Self‐management skills to improve mood	100%	100%
Lin et al. (2011)	None	Physical activity	100%	100%

Based on the JITAIs approach and our analysis, we adapted the TIDieR checklist to facilitate transparency in reporting JITAIs or personalized interventions alike, aiding future researchers to identify and evaluate essential information. Our adapted checklist (Table 3) expanded items 2, 6, 7 and 9. Intervention rationale (item 2) included four sub‐items, consisting of research purposes, research design, target behaviours and target population. Delivery mode (item 6) had three more sub‐items, covering data capturing, processing methods and outcome measures. Delivery setting (item 7) included three sub‐items related to research ethics, data protection methods and data sharing policies. Tailoring (item 9) was expanded to include six JITAI components, namely decision points, tailoring variables, intervention options, decision rules, proximal outcomes and distal outcome (Nahum‐Shani et al., 2018). An example using the checklist was provided in Appendix S9.

**TABLE 3:** JITAIs reporting checklist.

Categories	Items		Guidance	Checkbox
Brief name: what is the name of the intervention?	Item 1	Intervention type	Describe the intervention design as just‐in‐time adaptive intervention	
Why: why was the intervention developed, and why was the study conducted?	Item 2	Intervention rationale	Describe any rationale, theory or goal of the elements essential to the intervention	
	Item 2–1	Research purposes	Provide a statement of the aims or objectives behind the research being conducted	
	Item 2–2	Research design	Outline the systematic plans used to conduct the research	
	Item 2–3	Target behaviours	State the behaviour(s) evaluated in the intervention	
	Item 2–4	Target population	State the participant's characteristics targeted by the intervention	
What: what intervention materials and procedures were undertaken?	Item 3	Intervention materials	Describe any physical or informational materials used in the intervention, including those provided to participants or used in intervention delivery or in training of intervention providers. Provide information on where the materials can be accessed (e.g., online appendix, URL)	
	Item 4	Intervention procedures	Describe each of the procedures, activities and/or processes used in the intervention, including any enabling or support activities	
Who provided: Who or what tools were used to provide the intervention?	Item 5	Intervention providers	For each category of intervention provider (e.g., psychologist, nursing assistant), describe their expertise, background and any specific training given. If the provider is a computerized system, describe relevant information of the system	
How: how the intervention was delivered, and how user data and outcomes were captured, processed and evaluated?	Item 6	Delivery mode	Describe the modes of delivery (e.g., face‐to‐face or by some other mechanism, such as internet or telephone) of the intervention and whether it was provided individually or in a group	
	Item 6–1	Data capturing methods	Detail the techniques and tools used to collect information for tailoring the intervention, including both self‐report and passive monitoring	
	Item 6–2	Data processing methods	Provide details on the specific techniques used for processing data, including their sources, the development process and the intended purposes and contexts for use. This description should clarify how the data was prepared and transformed prior to its use in informing intervention delivery	
	Item 6–3	Outcome measures	Detail the metrics or standardized tools used to assess the study objectives, ideally breaking down into subjective and objective measurements	
Where: where was the intervention provided, and how were ethical issues, data protection and sharing methods handled?	Item 7	Delivery setting	Describe the type(s) of location(s) where the intervention occurred, including any necessary infrastructure or relevant features	
	Item 7–1	Research ethics	Provide the ethical approvals and permission obtained, including the types and sources of the approvals	
	Item 7–2	Data protection methods	Describe the technical and administrative measures implemented to ensure the security and privacy of remote data collection and monitoring, including information on the regulations or guidelines followed	
	Item 7–3	Data sharing policies	Describe the policies and regulations followed for data sharing between researchers and participants, including any involvement of third parties	
When and how much: when and how often was the intervention delivered, and what was the duration and dosage of the intervention?	Item 8	Intervention dosage	Describe the number of times the intervention was delivered and over what period of time including the number of sessions, their schedule, and their duration, intensity or dose	
Tailoring: was the intervention tailored, titrated or adapted, and if so, how?	Item 9	Tailoring	If the intervention was planned to be personalized, titrated or adapted, describe any rationale, theory or goal essential to the tailoring variables	
	Item 9–1	Decision points	Explain the timing when an intervention decision is made, including frequency, time of the day, duration of the assessment process	
	Item 9–2	Tailoring variables	Detail the sources of data used in making an intervention decision, including both self‐reported and passively monitored information	
	Item 9–3	Intervention options	Describe the various types of support offered in tailored scenarios, organizing them into clear and understandable categories for better comprehension	
	Item 9–4	Decision rules	Detail the metrics and criteria used to determine the choice of intervention and its timing, providing a diagram to illustrate the decision‐making process if possible	
	Item 9–5	Proximal outcomes	Describe the target behaviour that the intervention aims to change, and how they are related to the overall desired outcome	
	Item 9–6	Distal outcomes	State the ultimate goal or primary clinical outcome of the intervention	
Modification: was the intervention procedure modified at any point, and if so, how?	Item 10	Intervention modification	If the intervention was modified during the course of the study, describe the changes (what, why, when and how)	
How well: was the adherence assessed, and if so, what methods were used to evaluate it and how well did it perform?	Item 11	Adherence plans	If intervention adherence or fidelity was assessed, describe how and by whom, and if any strategies were used to maintain or improve fidelity, describe them	
	Item 12	Adherence results	If intervention adherence or fidelity was assessed, describe the extent to which the intervention was delivered as planned	

## DISCUSSION

This scoping review systematically identified, examined and summarized the current state‐of‐art of JITAIs in behaviour change. Our analysis has observed common principles in the development, implementation and evaluation of JITAIs from a wide range of health‐related behaviours. These observations were based on their theoretical foundations, operational methods, outcome assessments and quality of intervention reporting. Although prior reviews have undertaken similar investigations in physical activity (Hardeman et al., 2019), substance use (Perski et al., 2022) and disease management (Oikonomidi et al., 2023), their focus was limited to a specific behaviour or context, with studies primarily using RCTs, and only examining four JITAI components—tailoring variables, decision points, decision rules and intervention options (Nahum‐Shani et al., 2018). Our findings provided a broader perspective drawn from different health‐related behaviours. Some were congruent with prior analyses in aspects such as theoretical foundations, data capturing methods, and intervention reporting, while others presented more nuanced details in outcome evaluation and data processing.

Integrating theory into the intervention development is considered an important step but can be challenging due to unclear guidelines and overlapping constructs (Michie, 2005; Michie & Prestwich, 2010). A previous review on physical activity (Hardeman et al., 2019) reported that only five JITAIs were based on theories, but it also observed four BCTs, including goal setting, prompts/cues, feedback on behaviour and action planning. Our review took a more inclusive approach to identifying any supporting evidence. Despite nearly 74% of JITAIs reporting relevant evidence, we found that the majority of that evidence was limited to informing intervention options (67%). As introduced in the beginning, a practical challenge is identifying scientific models capable of supporting the dynamics of multi‐component interventions like JITAIs. Existing JITAIs typically selected and integrated their evidence on a component‐by‐component basis, without considering the interplay among components and the impact of within‐person and time‐varying effects. Another equally critical challenge is theory translation. For example, Rabbi's team described their experience of converting operant conditioning theory into intervention strategies (e.g., motivational messages) aimed at enhancing adherence to daily self‐reporting (Rabbi et al., 2020). They emphasized that theory translation is an iterative process requiring feedback from target users. This is to ensure that the translated elements induce the desired actions and align with user values. mHealth technologies, which are capable of tracking real‐time changes and providing dynamic feedback, can support research to identify which theoretical constructs work and how they work within individuals over time. Such understanding can help explain JITAIs mechanisms and improve the development of effective personalized interventions.

Identifying optimal timing to assess individual needs and determining relevant factors for tailoring are critical for effective data collection in JITAIs. This requires using the appropriate methods and tools to capture the necessary information at the right time without overwhelming the users. Our review found that over half of JITAIs were solely dependent on self‐reported data, indicating a strong reliance on subjective assessments within the current JITAIs landscape. This mirrors a previous review where over 75% of their included JITAIs required active monitoring from either patients, health professionals or both (Oikonomidi et al., 2023). Subjective assessments offer the advantage of a richer user‐centred perspective and potentially foster awareness of unwanted behaviours or triggers. However, they can be limited by user burdens and recall biases. In contrast, passive monitoring reduces the need for active user input, but it may not fully capture the nuances of personal experiences that lead to particular behaviours. A prior review suggested that the choice between active versus passive assessments should consider whether the target users would benefit and how to ethically collect high‐quality data without infringing on user privacy or causing undue burden (Perski et al., 2022). Further research is needed in examining the balance between active and passive user engagement in relation to assessing real‐time needs, thereby improving the quality of data collection and user involvement.

Real‐time data processing enables JITAIs to execute decision rules that determine the appropriate timing to offer the right support. No prior reviews have fully explored how decision rules were operated. We assessed decision rules and data processing methods with a primary focus on whether readers could clearly understand the underlying logic and practical applications. Still, 44% of studies did not provide descriptions of their data processing techniques. It should be noted that the complexities involved in describing more advanced computations, such as machine learning models, may challenge replicability. An observation was made in another review where 43% of decision rules were unreplicable (Oikonomidi et al., 2023). This issue highlighted a gap in the current JITAI literature regarding reporting completeness. In addition, a review on smoking cessation observed that JITAIs predominantly used static if‐then rules without accounting for time variance and user availability or receptivity (Perski et al., 2022). Particularly, user receptivity was found to have significant associations with factors such as age, personality, device type, day/time, phone battery, phone interaction and location (Künzler et al., 2019). Another study suggested that using machine learning models could lead up to a 40% improvement in receptivity as compared to the control model that delivered at random times (Mishra et al., 2021). Our review identified seven studies using machine learning methods, with one explicitly using a reinforcement learning model (Wang et al., 2021). The application of reinforcement learning may improve JITAIs effectiveness as it adapts to changes in users' behaviours and environments, while also using group‐level data to speed the learning process and result in improved performance (Liao et al., 2019). In future research, incorporating advanced computations and addressing user receptivity may improve the balance between involvement and burden. This could also reduce the chances of delivering an intervention without enough evidence of its effectiveness.

Finally, our analysis observed that JITAIs appeared to be efficacious in control settings, but we recognized that the field is still evolving and requires large‐scale validation for its clinical efficacy and effectiveness. Similar to the past review (Perski et al., 2022), the main challenge of analysing these studies was the heterogeneity of outcome metrics, measurement units and assessment frequencies. Despite the positive perception towards personalized functionality, it was difficult to infer exactly how tailoring mechanisms contribute to these positive results and which one performs better than others. Developing a standardized set of metrics to evaluate multi‐component interventions like JITAIs may improve cross‐study comparisons and distinguish single‐component effects. While we agree with existing reviews that the JITAI landscape holds considerable room for advancement, our quality assessment of intervention reporting indicated a growing maturity in the transparency of intervention design. To build on this progress, our proposed JITAIs reporting checklist focuses on enhancing aspects such as tailoring, real‐time data capturing and processing, and data governance. These elements are critical for improving the evaluation processes in future research on personalized interventions.

### Strengths and limitations

The strengths of this scoping review include an in‐depth examination of JITAI components and their implementation across a wide range of health‐related behaviours, an evaluation of intervention reporting quality, and having developed an intervention reporting checklist to improve transparency. However, limitations need to be addressed. First, our search strategy and selection criteria were designed to focus on the relevance and immediacy of intervention delivery in the mHealth context. While it enabled us to include a broad range of health‐related behaviours, it is possible that relevant publications have emerged outside our strategy and after the conclusion of our review period. This may limit the breadth and currency of our analysis. Additionally, the assessment of intervention reporting clarity was influenced by whether studies identified their interventions as JITAIs. Those that did not were less likely to provide JITAI‐specific information. Our proposed checklist was developed to enhance reporting practices among researchers of personalized interventions and encourage the adoption of JITAIs as a guiding framework.

## CONCLUSION

This scoping review provides an in‐depth overview of the current JITAIs in behaviour change, highlighting considerations in theoretical foundations, data capturing and process methods, outcome assessments and the quality of intervention reporting. It observes improved transparency in intervention reporting, though clarity could be enhanced in more complex components, such as decision rules, to facilitate better evaluation and replication. A reporting checklist was proposed to further advance JITAI research.

## AUTHOR CONTRIBUTIONS

**Ting‐Chen Chloe Hsu:** Methodology; investigation; writing – original draft; writing – review and editing; formal analysis; data curation; conceptualization; visualization. **Pauline Whelan:** Conceptualization; supervision; writing – review and editing; methodology. **Julie Gandrup:** Writing – review and editing; validation. **Christopher J. Armitage:** Writing – review and editing; supervision; conceptualization; methodology. **Lis Cordingley:** Supervision; methodology; conceptualization; writing – review and editing. **John McBeth:** Conceptualization; methodology; validation; writing – review and editing; supervision; visualization.

## Supporting information


Appendices S1–S9.

## Data Availability

The data supporting the findings are available in the Appendix S1.
