# Supplementary material for: Personalized interventions for behaviour change: A scoping review of just‐in‐time adaptive interventions
Source: Br J Health Psychol. 2024 Nov 14;30(1):e12766. doi: 10.1111/bjhp.12766 (PMC11583291; doi:10.1111/bjhp.12766)
Supplement: Supplementary file 1 — Appendices S1–S9. [file BJHP-30-0-s001.docx]

# Supplementary information

## Appendix S1: Search terms

| **Themes** | **Search terms** |
| --- | --- |
| Just-in-time adaptive interventions | Just-in-time adaptive intervention*, JITAI, Ecological momentary intervention*, EMI, Dynamic tailor*, Real-time intervention* |
| Mobile health | Mobile health, mHealth, Smartphone*, Mobile phone*, Mobile app*, Smartphone app* |
| Behaviour change | Behavio* model, Behavio* change, Behavio* change theor*, Theoretical framework |
| Intervention methodology | Intervention optimization, Experimental design, Ecological momentary assessment, Micro-randomised trial, Intervention development, Intervention evaluation, Protocol |
| Outcomes evaluation | User experience, Usability, Acceptability, Engagement, Feasibility, Clinical outcome*, Clinical benefit*, Health outcome*, Randomized controlled trial, Clinical controlled trial |

## Appendix S2: Data charting items

| **Categories** | **Details** |
| --- | --- |
| Study details | First author, Year of publication, Location, Intervention type, Research purpose, Research design, Target condition, Target behaviours, Intervention app, Intervention duration, Participant characteristics (age, sex) |
| JITAI components | Decision points, Tailoring variables (self-reporting, passive monitoring), Decision rules, Intervention options, Proximal outcomes, Distal outcome |
| Behaviour change evidence | Supporting evidence, Evidence-informed JITAI components |
| Real-time data capturing and processing | Data capturing methods (self-reporting, passive monitoring), Data processing methods |
| Outcome evaluations | Methods of assessing feasibility, acceptability, usability |
| Effectiveness and efficacy | Intervention and control groups |
| Micro-randomised trials | Intervention options |

## Appendix S3: Supporting evidence for behaviour change

| **Included studies** | **Target behaviours** | **Supporting evidence** | **Translated components** |
| --- | --- | --- | --- |
| Wang, 2023 | Mood, physical activity, sleep | Behaviour change techniques | Intervention options |
| Yang, 2023 | Smoking cessation | No information | N/A |
| Beres, 2022, 2021 | Fruit and vegetable consumption, drinking, smoking, and sexual behaviour | Nudges | Intervention options |
| Carlozzi, 2022 | Physical activity, sleep hygiene, mood | Behavioural activation approach | Intervention options |
| Ismail, 2022 | Sedentary behaviour | Habit formation | Decision points Intervention options Proximal outcome |
| Juarascio, 2022 | Eating behaviour | Cognitive-behavioural therapy | Intervention options |
| Mair, 2022 | Physical activity | Behaviour Change Wheel | Intervention options |
| Morgiève, 2022 | Suicidal behaviour | No information | N/A |
| Sizemore, 2022 | Self-management skills | No information | N/A |
| Walters, 2022 | Alcohol use | No information | N/A |
| Blevins, 2021 | Alcohol use | Cognitive-behavioural therapy | Engagement |
| Coughlin, 2021 | Substance use | Operant conditioning | Intervention options |
| Fulford, 2021 | Social skills | Cognitive-behavioural therapy | Intervention options |
| Gire, 2021 | Self-management skills | Cognitive-behavioural therapy | Intervention options |
| Hawker, 2021 | Gambling behaviour | Cognitive-behavioural therapy  Motivational interviewing | Intervention options |
| Juarascio, 2021 [1] | Dietary behaviour | Cognitive-behavioural therapy for Bulimia Nervosa | Intervention options Tailoring variables Proximal outcomes |
| Juarascio, 2021 [2] | Dietary behaviour | Integrative Cognitive‐Affective Therapy | Intervention options |
| Santa Maria, 2021 | HIV risk behaviour | Information, Motivation, and Behavioural Skills model | Intervention app |
| Wang, 2021 | Physical activity | Behaviour Change Wheel | Intervention options |
| Conroy, 2020 | Fluid consumption | Negative reinforcement (operant conditioning) | Decision rules |
| Hébert, 2020 | Smoking cessation | No information | N/A |
| Low, 2020 | Sedentary behaviour | No information | N/A |
| NeCamp, 2020 | Mood, physical activity, sleep | Fogg behaviour model  Motivational interviewing | Intervention options |
| Scott, 2020 | Substance use risk behaviour | Self-determination theory | No information |
| Shrier, 2020 | High risk sexual behaviour | Behaviour-Determinant-Intervention Logic Model Motivational interviewing Cognitive-behavioural therapy | Intervention design Goal identification Intervention options |
| Valle, 2020 | Dietary behaviour | Behaviour change techniques | Intervention options |
| Bartlett Ellis, 2019 | Treatment adherence | Systems thinking  Habit formation | Intervention options |
| Forman, 2019 [1, 2] | Dietary behaviour | Behaviour change techniques | Intervention options |
| Hiremath, 2019 | Physical activity | Operant conditioning | Intervention options |
| Hoeppner, 2019 | Smoking cessation | Behaviour change techniques | Intervention options |
| Klasnja, 2019 | Physical activity | No information | N/A |
| Levin, 2019 [1, 2] | Self-management skills | Acceptance and commitment therapy | Intervention options |
| O’Donnell, 2019 | Alcohol use | Protective Behavioural Strategies | Intervention options |
| Hébert, 2018 | Smoking cessation | No information | N/A |
| Shrier, 2018, 2014 | Marijuana use | Motivational enhancement therapy | Intervention options |
| Van Dantzig, 2018 | Physical activity | No information | N/A |
| Attwood, 2017 | Alcohol use | Behaviour change techniques | Intervention app Intervention options |
| Leonard, 2017 | Alcohol use | Motivational interviewing Cognitive-behavioural therapy | Intervention options |
| Businelle, 2016 | Smoking cessation | No information | N/A |
| Ding, 2016 | Physical activity | Fogg behaviour model  Goal setting theory  Habit formation | Tailoring variables  Intervention options  Decision rules |
| Naughton, 2016 | Smoking cessation | Learning theory Taxonomy of smoking behaviour change techniques | Intervention options |
| Wenze, 2016 | Treatment adherence | Psychoeducational and cognitive-behavioural principles | Intervention options |
| Depp, 2015 | Self-management skills | Psychoeducational approaches | Intervention options |
| Finkelstein, 2015 | Sedentary behaviour | No information | N/A |
| Gonzalez, 2015 | Alcohol use | Motivational interviewing (MI) Relapse prevention model Community reinforcement approach Psychoeducational approaches | Intervention options Decision rules |
| Ingersoll, 2015, 2014 | Treatment adherence | Information, Motivation and Behaviour Skills (IMB) Model of Adherence Social Action Theory (SAT) | Tailoring variables Intervention options |
| Mundi, 2015 | Dietary behaviour | No information | N/A |
| Pellegrini, 2015 | Sedentary behaviour | No information | N/A |
| Rabbi, 2015 [1, 2] | Physical activity and dietary behaviour | Learning theory  Social cognitive theory Fogg behaviour model | Intervention options |
| Thomas, 2015 | Sedentary behaviour | No information | N/A |
| Ben-Zeev, 2014 | Self-management skills | Cognitive model of psychosis Stress-vulnerability model of schizophrenia Evidence-based interventions (i.e., cognitive restructuring, behavioural tailoring, social skills training, illness management and recovery, anger management, behavioural activation, sleep hygiene) | Tailoring variables Decision rules Intervention options |
| Dulin, 2014 | Alcohol use | Motivational interviewing (MI) Relapse prevention model Community reinforcement approach Combined Behavioural Intervention Manual | Intervention options Tailoring variables Decision rules |
| Gustafson, 2014 | Alcohol use | Self-determination theory | No information |
| Van Dantzig, 2013 | Sedentary behaviour | No information | N/A |
| Burns, 2011 | Self-management skills | Behavioural activation approach | Intervention options |
| Lin, 2011 | Physical activity | No information | N/A |

## Appendix S4: Real-time data capturing and process methods

| **Included studies** | **Target behaviours** | **Data capturing methods** | **Data processing methods** |
| --- | --- | --- | --- |
| Wang, 2023 | Mood, physical activity, sleep | **Self-report**: daily prompts for mood  **Passive monitoring:** wearable devices Fitbit or Apple Watch for sleep and step counts | No information |
| Yang, 2023 | Smoking cessation | **Passive monitoring**: four sensors for physiological responses - a chest band/box with two electrodes to assess electrocardiography and respiration, and two wrist sensors to assess arm movement | cStress algorithm uses physiological signature to detect stress and respiration data and wrist movements to detect smoking behaviour |
| Beres, 2022, 2021 | Fruit and vegetable consumption, drinking, smoking, and sexual behaviour | **Self-report**: EMA  **Self-initiated event-contingent report** (if engage with one of the five study behaviours) | No information |
| Carlozzi, 2022 | Physical activity, sleep hygiene, mood | **Self-report**: HRQOL ratings **Passive monitoring**: activity tracker (Fitbit) | No information |
| Ismail, 2022 | Sedentary behaviour | **Passive monitoring**: smartphone | Researcher developed decision-making flowchart |
| Juarascio, 2022 | Dietary behaviour | **Passive monitoring**: CGM  **Self-initiated report** | SenseSupport uses a meal detection algorithm to detect meal consumption and estimate the size and macronutrient content of a meal |
| Mair, 2022 | Physical activity | **Passive monitoring**: activity tracker (Fitbit), smartphone | Algorithm used to deliver personalised JITAI messages was developed with the Firebase Admin Python package, and an open access Fitbit API package |
| Morgiève, 2022 | Suicidal behaviour | **Self-report**: EMA  **Self-initiated report** | A decision tree developed by focus group |
| Sizemore, 2022 | Self-management skills | **Self-report**: EMA | No information |
| Walters, 2022 | Alcohol use | **Self-report**: EMA  **Self-initiated report** | A predictive machine learning model |
| Blevins, 2021 | Alcohol use | **Self-report**: EMA  **Self-initiated report** | No information |
| Coughlin, 2021 | Substance use | **Self-report**: daily survey | The iRTT system includes feedback loops monitoring symptoms and a machine learning algorithm to provide personalised intervention |
| Fulford, 2021 | Social skills | **Self-report**: EMA | No information |
| Gire, 2021 | Self-management skills | **Self-report**: ESM | CBT-based algorithm |
| Hawker, 2021 | Gambling behaviour | **Self-report**: EMA | No information |
| Juarascio, 2021 [1] | Dietary behaviour | **Self-initiated report** | A theory-based algorithm detecting risk of engaging eating disorder behaviour |
| Juarascio, 2021 [2] | Dietary behaviour | **Self-report**: EMA  **Self-initiated report** | Researcher developed algorithm to detect sexual behaviour and drug use risks |
| Santa Maria, 2021 | HIV risk behaviour | **Self-report**: EMA | A reinforcement Learning model |
| Wang, 2021 | Physical activity | **Passive monitoring**: smartphone | No information |
| Conroy, 2020 | Fluid consumption | **Passive monitoring**: H2Opal-connected water bottle, activity tracker (Fitbit) | Algorithms detecting drinking behaviour and availability |
| Hébert, 2020 | Smoking cessation | **Self-report**: EMA  **Self-initiated report** | An algorithm detecting risk of smoking lapse |
| Low, 2020 | Sedentary behaviour | **Self-report**: daily symptom ratings  **Passive monitoring**: activity tracker (Fitbit) | No information |
| NeCamp, 2020 | Mood, physical activity, sleep | **Self-report**: EMA for mood  **Passive monitoring**: activity tracker for sleep and step counts (Fitbit) | No information |
| Scott, 2020 | Risk behaviour for substance use | **Self-report**: EMA | No information |
| Shrier, 2020 | High risk sexual behaviour | **Self-report**: EMA | No information |
| Valle, 2020 | Dietary behaviour | **Self-report**: self-monitoring diet form  **Passive monitoring**: wireless scale and activity tracker (Fitbit) | An algorithm that randomised intervention messages at a 50% rate |
| Bartlett Ellis, 2019 | Treatment adherence | **Self-report**: a smart button  **Fully self-initiated** | A cloud-based server with a computer algorithm containing text messages |
| Forman, 2019 [1, 2] | Dietary behaviour | **Self-report**: dietary lapses and triggers update  **Self-initiated report** | Machine learning algorithms use a combination of group- and individual-level data to predict lapse risk |
| Hiremath, 2019 | Physical activity | **Self-report**: EMA  **Passive monitoring**: a wrist-worn smartwatch, a Bluetooth-based wheel rotation monitor | The PHIRE app used aC4.5 decision tree machine-learning algorithm to classify wheelchair-based PAs and estimate PA levels once per minute |
| Hoeppner, 2019 | Smoking cessation | **Self-report**: EMA, text-message  **Self-initiated report** | No information |
| Klasnja, 2019 | Physical activity | **Passive monitoring**: smartphone, activity tracker (Jawbone Up Move) | Developing algorithms |
| Levin, 2019 [1, 2] | Self-management skills | **Self-report**: EMA | No information |
| O’Donnell, 2019 | Alcohol use | **Self-report**: self-monitoring survey | No information |
| Hébert, 2018 | Smoking cessation | **Self-report**: EMA  **Self-initiated report** | An algorithm detecting risk of smoking lapse |
| Shrier, 2018 | Marijuana use | **Self-report**: EMA | No information |
| Van Dantzig, 2018 | Physical activity | **Passive monitoring**: a wrist-worn activity tracker (Philips Health Watch), a location-tracking app, Bluetooth-enabled iBeacons | The remote coaching engine selected the appropriate coaching messages from the message database |
| Attwood, 2017 | Alcohol use | **Passive monitoring**: smartphone (location) | No information |
| Leonard, 2017 | Alcohol use | **Self-report**: CBT-informed questions  **Self-initiated report**  **Passive monitoring**: electrodermal activity (EDA, Empatica E4) | No information |
| Businelle, 2016 | Smoking cessation | **Self-report**: EMA  **Self-initiated report** | An algorithm detecting risk of smoking lapse |
| Ding, 2016 | Physical activity | **Passive monitoring**: smartphone watch and smartphone | WalkMore detects the first three moments using app usage api and activity recognition api (walking, running, driving and still) provided by the Android Platform |
| Naughton, 2016 | Smoking cessation | **Self-initiated report**  **Passive monitoring during the second stage**: smartphone | No information |
| Wenze, 2016 | Treatment adherence | **Self-report**: questions of bipolar disorder symptoms | No information |
| Depp, 2015 | Self-management skills | **Self-report:** SMS survey | We developed a mobile enabled web-based program to deliver personalized questionnaires capable of delivering pre-programmed interactive algorithm-based responses based on symptoms or early warning signs reported |
| Finkelstein, 2015 | Sedentary behaviour | **Passive monitoring:** activity tracker (Fitbit) | Monitoring and text-message algorithms are developed with #C (POSTed XML) and Fitbit API |
| Gonzalez, 2015 | Alcohol use | **Self-report**: app survey  **self-initiated report**  **Passive monitoring**: smartphone (location) | No information |
| Ingersoll, 2015 | Treatment adherence | **Self-report**: text query | SMS system |
| Mundi, 2015 | Dietary behaviour | **Self-report**: EMA | Researcher developed algorithm |
| Pellegrini, 2015 | Sedentary behaviour | **Self-report**: app prompt  **Passive monitoring**: waist-worn accelerometer (Shimmer) | No information |
| Rabbi, 2015 [1, 2] | Physical activity and dietary behaviour | **Self-report**: smartphone  **Passive monitoring**: smartphone | A sequential decision making algorithm, Multiarmed Bandit, to generate suggestions (exploit-explore strategy) |
| Thomas, 2015 | Sedentary behaviour | **Passive monitoring**: smartphone | Pre-validated algorithm detecting sedentary behaviour |
| Ben-Zeev, 2014 | Self-management skills | **Self-report**: daily prompts through smartphone app | The primary FOCUS app uses interactive algorithms to generate brief assessments |
| Dulin, 2014 | Alcohol use | **Self-report**: app survey  **self-initiated report**  **Passive monitoring**: smartphone (location) | No information |
| Gustafson, 2014 | Alcohol use | **Passive monitoring**: smartphone (location) | No information |
| Ingersoll, 2014 | Treatment adherence | **Self-report**: text query | SMS system |
| Shrier, 2014 | Marijuana use | **Self-report**: momentary self-monitoring | No information |
| Van Dantzig, 2013 | Sedentary behaviour | **Passive monitoring**: smartphone, commercial activity monitor, software to measure keyboard and mouse activities | SMS algorithm |
| Burns, 2011 | Self-management skills | **Self-report**: EMA | Machine learning (regression trees, J48 classifiers) |
| Lin, 2011 | Physical activity | **Passive monitoring**: smartphone | Motivate system consists of Motivate service, Motivate web application and Mobile API which communicates with Motivate mobile application. |

## Appendix S5: Outcome evaluations

| **Included studies** | **Target behaviours** | **Feasibility** | **Acceptability** | **Usability** |
| --- | --- | --- | --- | --- |
| Wang, 2023 | Mood, physical activity, sleep | No information | No information | No information |
| Yang, 2023 | Smoking cessation | Retention of in-person visits. Adherence to the use of sensors, completion of strategies and EMAs, and nicotine patch use. The difficulty of using the smartphone and wearable sensors and the number of days it took to be comfortable wearing the sensors. | Client Satisfaction Questionnaire. A questionnaire on the utility of mindfulness strategies. Mindfulness strategy ratings. Participants’ feedback on the treatment using open-ended questions. Items queried the most and least favourite parts of treatment, barriers to using mindfulness, and general suggestions for improvement of the treatment. | System Usability Scale |
| Beres, 2022 | Fruit and vegetable consumption, drinking, smoking, and sexual behaviour | Access to electricity, cellular reception, daily and weekly prompt response rates, prompt-driven behavioural self-report (daily, weekly and self-initiated responses), geospatial data collection, reported phone and app use problems | Daily and weekly prompt response rates, prompt-driven behavioural self-report (daily, weekly and self-initiated responses), perceptions of EMAI participation (asked at day 90 about like/dislike) | No information |
| Carlozzi, 2022 | Physical activity, sleep hygiene, mood | Intervention compliance, study-specific questionnaire | Study-specific questionnaire | No information |
| Ismail, 2022 | Sedentary behaviour | No information | No information | The Mobile Application Rating Scale, semi-structured interview and app usage for user engagement |
| Juarascio, 2022 | Dietary behaviour | CGM data, weekly therapist and participant questionnaires, and retention calculations. | Participant feedback questionnaire and interviews, including comfort, pain, disruption of daily life, ease of use, and helpfulness of the SenseSupport system. | No information |
| Mair, 2022 | Physical activity | Recruitment and retention, intervention delivery, data collection and missing data | Satisfaction with research process, technological components (intervention), app components, intervention (user experience) | No information |
| Morgiève, 2022 | Suicidal behaviour | No information | Completion rate, Mobile App Rating Scale | No information |
| Sizemore, 2022 | Self-management skills | EMA completion, percent of days participants reported stress, number of intervention activities which were triggered, percentage of intervention activities completed, percentage of positive messages viewed, and the average amount of time on demand content was accessed. | Questionnaire, interview, Client Satisfaction Questionnaire 8, Information Systems Success Model | System Usability Scale |
| Walters, 2022 | Alcohol use | No information | No information | Usefulness: number of prompts, tips features, helpfulness, interesting |
| Beres, 2021 | Fruit and vegetable consumption, drinking, smoking, and sexual behaviour | Completeness of data collection | No information | No information |
| Blevins, 2021 | Alcohol use | Recruitment rates, EMA response rates, follow-up rates, and qualitative interviews | Recruitment rates, EMA response rates, follow-up rates, and qualitative interviews | No information |
| Coughlin, 2021 | Substance use | Self-reported engagement points | Questionnaires of appearance, enjoyment, content, data acceptability, technical issues | No information |
| Fulford, 2021 | Social skills | No information | No information | No information |
| Gire, 2021 | Self-management skills | Recruitment rates, interviews | Engagement, app usage, interviews | Interviews |
| Hawker, 2021 | Gambling behaviour | Retention rates, app use metrics (compliance), intervention use | Helpfulness via separate 11-point Visual Analogue Scales. Satisfaction via the 3-item Client-Satisfaction Questionnaire-3. Impact of the intervention on participants’ awareness, knowledge, attitude, intention to change, help-seeking behaviour, and behaviour change in relation to gambling cravings via the 6-item App-Specific subscale of the Mobile App Rating Scale. | No information |
| Juarascio, 2021 [1] | Dietary behaviour | App usage, user adherence, weekly questionnaire | Usefulness scale, weekly questionnaires, interviews | No information |
| Juarascio, 2021 [2] | d | App usage, including the number of survey prompts delivered and completed, as well as the number of risk alerts delivered and opened | Technology Acceptance Model Scales, text-entry questions | No information |
| Santa Maria, 2021 | HIV risk behaviour | No information | No information | No information |
| Wang, 2021 | Physical activity | No information | No information | Number of reminders reacted, number of physical activities performed, questionnaire, interviews |
| Conroy, 2020 | Fluid consumption | No information | Questionnaires of perceived value (The User Burden Scale), satisfaction, barriers to adherence, experienced automaticity of fluid consumption (Behavioural Automaticity Scale from the Self-Report Habit Index) | The System Usability Scale |
| Hébert, 2020 | Smoking cessation | Engagement and compliance to EMA | Surveys on satisfaction | No information |
| Low, 2020 | Sedentary behaviour | Accrual and retention rates, compliance with reporting symptoms and Daily compliance with wearing the watch | No information | Weekly ratings on satisfaction, easiness and pleasantness; The System Usability Scale |
| NeCamp, 2020 | Mood, physical activity, sleep | No information | No information | No information |
| Scott, 2020 | Risk behaviour for substance use | No information | No information | No information |
| Shrier, 2020 | High risk sexual behaviour | Duration and retention rate of counselling session, number of EMI report and survey | Surveys on perceived utility and usefulness of counselling sessions and the app | No information |
| Valle, 2020 | Weight management behaviour | No information | No information | App usage for engagement |
| Bartlett Ellis, 2019 | Treatment adherence | Recruitment rates, the number of times the mHealth set up, concordance, willingness to use the system | Qualitative feedback provided by participants (thoughts on whether the SMS text messages were helpful to support medication taking) | No information |
| Forman, 2019 [1] | Dietary behaviour | End-of-day survey question about that day’s risk alert utility and accuracy and perceived helpfulness | The Technology Acceptance Model Scales to assess satisfaction, perceived usefulness and usability, and technical problems | Number of survey prompts delivered and completed, and number of risk alerts received and viewed |
| Forman, 2019 [2] | Dietary behaviour | No information | The Technology Acceptance Model Scales were used to assess participant satisfaction with and acceptance of the app-based interventions at posttreatment, including ease of use and usefulness of the app | No information |
| Hiremath, 2019 | Physical activity | No information | An exit interview for ease of use, any challenges they faced with the system, what they thought about the PA level feedback and JITAI notifications, and if they would be interested in continuing to use the JITAI system and/or recommend it to their friends | No information |
| Hoeppner, 2019 | Smoking cessation | No information | SmokefreeTXT usage data, review of messages for helpfulness | No information |
| Klasnja, 2019 | Physical activity | No information | No information | No information |
| Levin, 2019 [1] | Self-management skills | No information | Survey for satisfaction | The System Usability Scale; App usage for engagement |
| Levin, 2019 [2] | Self-management skills | No information | No information | No information |
| O’Donnell, 2019 | Alcohol use | The frequency of applying PBS, adherence with the app | Interview based on the Enlight measure. | No information |
| Hébert, 2018 | Smoking cessation | No information | No information | No information |
| Shrier, 2018 | Marijuana use | Monthly recruitment and enrolment rates, retention rates, momentary and daily response rates | Agreement on a 5-point Likert-type scale to 21 items for helpfulness, and session quality. Acceptability of procedures, overall study usefulness, most and least favourite parts of the study, what they would change, and any additional comments | No information |
| Van Dantzig, 2018 | Physical activity | No information | No information | Semi-structured interview, transcribed and labeled as positive, negative or neutral |
| Attwood, 2017 | Alcohol use | No information | No information | App usage patterns and user satisfaction. Semi-structured interview for any questions or issues and to explore users’ experiences of the app, perceptions of usability, acceptability, effectiveness and to obtain suggestions for improvements to this product |
| Leonard, 2017 | Alcohol use | App usage, including the frequency of reaching their EDA threshold, the number and content of reports made on the app (sensorband-triggered and self-initiated), and the amount of time spent on each screen | The Client Satisfaction Questionnaire, additional surveys, and semi-structured interview to explore participants’ perceptions of the technology, including the challenges, and the barriers and facilitators of using it in their daily life to help them achieve their goals | No information |
| Businelle, 2016 | Smoking cessation | Survey on usefulness | Survey on satisfaction | No information |
| Ding, 2016 | Physical activity | No information | Acceptance of reminder | System usability scale to measure the usability of the system |
| Naughton, 2016 | Smoking cessation | Mean frequency and location of smoking reports, current location (ie, background samples), proportion of EoD surveys completed, proportion of days where EoD smoking category exceeded daily smoking reports, proportion of smoking reports with geolocation data, accuracy of geolocation generated by the device, number of active geofences created per participant, proportion of participants receiving any geofence-triggered support messages, the number of geofence support messages delivered, the time to view geofence support messages, and the proportion of support messages rated. | No information | Interview |
| Wenze, 2016 | Treatment adherence | EMI usage | The Client Satisfaction Questionnaire, additional survey and open-ended interview | No information |
| Depp, 2015 | Self-management skills | Compliance | A 10-point satisfaction and helpfulness scales. Specific acceptability of mobile devices focused on both positive and negative elements rated on a 5-point scale | No information |
| Finkelstein, 2015 | Sedentary behaviour | No information | No information | No information |
| Gonzalez, 2015 | Alcohol use | No information | No information | No information |
| Ingersoll, 2015 | Treatment adherence | Enrolment and retention, response rate with app | Satisfaction | No information |
| Mundi, 2015 | Dietary behaviour | Education modules being completed | Survey for satisfaction | No information |
| Pellegrini, 2015 | Sedentary behaviour | NEAT! application and intervention accelerometer usage | A 16-item questionnaire and a brief 14-question interview for acceptability. The questionnaire and interviews addressed current perception, liking, barriers, and future use of the application and intervention accelerometer | No information |
| Rabbi, 2015 [1] | Physical activity and dietary behaviour | Adherence | A suggestion-rating survey to evaluate user intentions to follow the suggestions | Daily diary and In-depth semi-structured interviews measured participant feedback regarding the suggestions |
| Rabbi, 2015 [2] | Physical activity and dietary behaviour | No information | A suggestion-rating survey to evaluate user intentions to follow the suggestions | No information |
| Thomas, 2015 | Sedentary behaviour | No information | No information | Engagement was evaluated by the number of days, and hours per day, that the smartphone was powered on and carried by the participant in a pocket or via the provided clip |
| Ben-Zeev, 2014 | Self-management skills | Recruitment and retention rates, app usage | A 26-item self-report acceptability/usability measure comprised of adapted items from the System Usability Scale, System Usability Questionnaire, Technology Assessment Model, Measurement Scales and Usefulness, Satisfaction, and Ease questionnaire | A 26-item self-report acceptability/usability measure comprised of adapted items from the System Usability Scale, System Usability Questionnaire, Technology Assessment Model, Measurement Scales and Usefulness, Satisfaction, and Ease questionnaire |
| Dulin, 2014 | Alcohol use | No information | No information | Questionnaires related to system usability, perceived helpfulness and ease of use. Interview about helpfulness for each feature. |
| Gustafson, 2014 | Alcohol use | No information | No information | No information |
| Ingersoll, 2014 | Treatment adherence | Response rates by day | Self-report of their acceptance and liking | Usability testing for message use |
| Shrier, 2014 | Marijuana use | Recruitment and retention rates | Feedback on study burden and usefulness | No information |
| Van Dantzig, 2013 | Sedentary behaviour | No information | A semi-structured interview for experiences with SitCoach, their current sedentary break habits and their awareness of the importance of breaks from sitting. | Two questionnaires for usability of the application and perceived locus of control with respect to reducing sitting time |
| Burns, 2011 | Self-management skills | Intervention adherence, app use and website use | Semi-structured interview | No information |
| Lin, 2011 | Physical activity | User feedback | No information | User feedback and interview |

## Appendix S6: Intervention and control groups in efficacy and effectiveness studies

| **Included studies** | **Research purposes** | **Research designs** | **Target behaviours** | **Intervention/control groups** |
| --- | --- | --- | --- | --- |
| Wang, 2023 | Effectiveness | MRT | Mood, physical activity, sleep | Randomised delivery (daily at 3pm):  Received a push notification (50%)  No push notification (50%)  Randomised competition (weekly):  Competition arm (50%)  Non-competition arm (50%)  Randomised competition type (weekly):  Sleep (50%)  Step count (50%)  Randomised competition opponent (weekly):  Total randomisation (33%)  Within the same institution (33%)  Within the same speciality (33%) |
| Ismail, 2022 | Effectiveness | Mixed-methods between group design | Sedentary behaviour | Intervention: JITAIs Control: static feedback |
| Beres, 2021 | Feasibility  Estimate effect | Pilot RCT | Fruit and vegetable consumption, drinking, smoking, and sexual behaviour | Intervention: JITAIs  Control: EMA |
| Santa Maria, 2021 | Efficacy | Pilot RCT | HIV risk behaviour | Intervention: JITAIs Control: static feedback |
| Hébert, 2020 | Feasibility  Preliminary efficacy | Pilot RCT | Smoking cessation | Intervention: JITAIs  Control: QuitGuide app, usual tobacco cessation clinic care |
| Forman, 2019 [2] | Efficacy | RCT | Dietary behaviour | Weight Watchers + OnTrack: JITAIs Weight Watchers: daily dietary goal |
| Klasnja, 2019 | Efficacy | MRT | Physical activity | Randomised delivery: 40% no suggestion 30% walking suggestion 30% anti-sedentary suggestion |
| Levin, 2019 [1] | Efficacy | RCT | Self-management skills | Intervention: JITAIs Control: randomised skill coaching |
| Levin, 2019 [2] | In-the-moment effects | RCT | Self-management skills | Intervention: JITAIs Control: randomised skill coaching |
| Van Dantzig, 2018 | Effectiveness and user experience | RCT | Physical activity | Intervention: JITAI (context-aware condition) - suggestion or feedback based on sensor data  Control: general advice message |
| Finkelstein, 2015 | Potential efficacy | Randomized crossover design | Sedentary behaviour | "Group A: reminders (4 weeks) + no reminders (4 weeks)  Group B: no reminders (4 weeks) + reminders (4 weeks)" |
| Ingersoll, 2015 | Preliminary efficacy | Pilot RCT | Treatement adherence | "Intervention: JITAI (TEXT)  Control: usual care" |
| Thomas, 2015 | Intervention effect | Randomised counterbalanced design | Sedentary behaviour | Randomised walking breaks: 3-min after 30 SB min 6-min after 60 SB min 12-min after 120 SB min |
| Gustafson, 2011 | Effectiveness | Un-blinded randomized controlled trial | Alcohol use | "Intervention: JITAI (A-CHESS)  Control: treatment as usual" |

## Appendix S7: Assessment of the TIDieR checklist

| **Included studies** | **1** | **2** | **3** | **4** | **5** | **6** | **7** | **8** | **9** | **10** | **11** | **12** |
| --- | --- | --- | --- | --- | --- | --- | --- | --- | --- | --- | --- | --- |
| Wang, 2023 | Yes | Yes | Yes | Yes | Yes | Yes | Yes | Yes | Yes | N/A | Yes | Yes |
| Yang, 2023 | Yes | Yes | Yes | Yes | Yes | Yes | Yes | Yes | Yes | Yes | Yes | Yes |
| Beres, 2021, 2022 | Yes | Yes | Yes | Yes | Yes | Yes | Yes | Yes | Yes | N/A | Yes | Yes |
| Carlozzi, 2022 | Yes | Yes | Yes | Yes | Yes | Yes | Yes | Yes | Yes | N/A | Yes | Yes |
| Ismail, 2022 | Yes | Yes | Yes | Yes | Yes | Yes | Yes | Yes | Yes | N/A | Yes | Yes |
| Juarascio, 2022 | Yes | Yes | Yes | Yes | Yes | Yes | Yes | Yes | Yes | N/A | Yes | Yes |
| Mair, 2022 | Yes | Yes | Yes | Yes | Yes | Yes | Yes | Yes | Yes | N/A | Yes | Yes |
| Morgiève, 2022 | Yes | Yes | Yes | Yes | Yes | Yes | Yes | Yes | Yes | N/A | Yes | Yes |
| Sizemore, 2022 | Yes | Yes | Yes | Yes | Yes | Yes | Yes | Yes | Yes | N/A | Yes | Yes |
| Walters, 2022 | Yes | Yes | Yes | Yes | Yes | Yes | Yes | Yes | Yes | N/A | Yes | Yes |
| Blevins, 2021 | Yes | Yes | Yes | Yes | Yes | Yes | Yes | Yes | Yes | N/A | Yes | Yes |
| Coughlin, 2021 | Yes | Yes | Yes | Yes | Yes | Yes | Yes | Yes | Yes | N/A | Yes | Yes |
| Fulford, 2021 | Yes | Yes | Yes | Yes | Yes | Yes | Yes | Yes | Yes | N/A | Yes | Yes |
| Gire, 2021 | Yes | Yes | Yes | Yes | Yes | Yes | Yes | Yes | Yes | N/A | Yes | Yes |
| Hawker, 2021 | Yes | Yes | Yes | Yes | Yes | Yes | Yes | Yes | Yes | N/A | Yes | Yes |
| Juarascio, 2021 [1] | Yes | Yes | Yes | Yes | Yes | Yes | Yes | Yes | Yes | N/A | Yes | Yes |
| Juarascio, 2021 [2] | Yes | Yes | Yes | Yes | Yes | Yes | Yes | Yes | Yes | N/A | Yes | Yes |
| Santa Maria, 2021 | Yes | Yes | Yes | Yes | Yes | Yes | Yes | Yes | Yes | N/A | Yes | Yes |
| Wang, 2021 | Yes | Yes | Yes | Yes | Yes | Yes | Yes | Yes | Yes | N/A | Yes | Yes |
| Conroy, 2020 | Yes | Yes | Yes | Yes | Yes | Yes | Yes | Yes | Yes | N/A | Yes | Yes |
| Hébert, 2020 | Yes | Yes | Yes | Yes | Yes | Yes | Yes | Yes | Yes | N/A | Yes | Yes |
| Low, 2020 | Yes | Yes | Yes | Yes | Yes | Yes | Yes | Yes | Yes | N/A | Yes | Yes |
| NeCamp, 2020 | Yes | Yes | Yes | Yes | Yes | Yes | Yes | Yes | Yes | N/A | Yes | Yes |
| Scott, 2020 | Yes | Yes | Yes | Yes | Yes | Yes | Yes | Yes | Yes | N/A | Yes | Yes |
| Shrier, 2020 | Yes | Yes | Yes | Yes | Yes | Yes | Yes | Yes | Yes | N/A | Yes | Yes |
| Valle, 2020 | Yes | Yes | Yes | Yes | Yes | Yes | Yes | Yes | Yes | N/A | Yes | Yes |
| Bartlett Ellis, 2019 | Yes | Yes | Yes | Yes | Yes | Yes | Yes | Yes | Yes | N/A | Yes | Yes |
| Forman, 2019 [1, 2] | Yes | Yes | Yes | Yes | Yes | Yes | Yes | Yes | Yes | Yes | Yes | Yes |
| Hiremath, 2019 | Yes | Yes | Yes | Yes | Yes | Yes | Yes | Yes | Yes | N/A | Yes | Yes |
| Hoeppner, 2019 | Yes | Yes | Yes | Yes | Yes | Yes | Yes | Yes | Yes | N/A | Yes | Yes |
| Klasnja, 2019 | Yes | Yes | Yes | Yes | Yes | Yes | Yes | Yes | Yes | N/A | Yes | Yes |
| Levin, 2019 [1, 2] | Yes | Yes | Yes | Yes | Yes | Yes | Yes | Yes | Yes | N/A | Yes | Yes |
| O’Donnell, 2019 | Yes | Yes | Yes | Yes | Yes | Yes | Yes | Yes | Yes | N/A | Yes | Yes |
| Hébert, 2018 | Yes | Yes | Yes | Yes | Yes | Yes | Yes | Yes | Yes | N/A | Yes | Yes |
| Shrier, 2018, 2014 | Yes | Yes | Yes | Yes | Yes | Yes | Yes | Yes | Yes | N/A | Yes | Yes |
| Van Dantzig, 2018 | Yes | Yes | Yes | Yes | Yes | Yes | Yes | Yes | Yes | N/A | Yes | Yes |
| Attwood, 2017 | Yes | Yes | Yes | Yes | Yes | Yes | Yes | Yes | Yes | N/A | Yes | Yes |
| Leonard, 2017 | Yes | Yes | Yes | Yes | Yes | Yes | Yes | Yes | Yes | N/A | Yes | Yes |
| Businelle, 2016 | Yes | Yes | Yes | Yes | Yes | Yes | Yes | Yes | Yes | N/A | Yes | Yes |
| Ding, 2016 | Yes | Yes | Yes | Yes | Yes | Yes | Yes | Yes | Yes | N/A | Yes | Yes |
| Naughton, 2016 | Yes | Yes | Yes | Yes | Yes | Yes | Yes | Yes | Yes | N/A | Yes | Yes |
| Wenze, 2016 | Yes | Yes | Yes | Yes | Yes | Yes | Yes | Yes | Yes | N/A | Yes | Yes |
| Depp, 2015 | Yes | Yes | Yes | Yes | Yes | Yes | Yes | Yes | Yes | N/A | Yes | Yes |
| Finkelstein, 2015 | Yes | Yes | Yes | Yes | Yes | Yes | Yes | Yes | Yes | N/A | Yes | Yes |
| Gonzalez, Dulin, 2015, 2014 | Yes | Yes | Yes | Yes | Yes | Yes | Yes | Yes | Yes | N/A | Yes | Yes |
| Ingersoll, 2015, 2014 | Yes | Yes | Yes | Yes | Yes | Yes | Yes | Yes | Yes | N/A | Yes | Yes |
| Mundi, 2015 | Yes | Yes | Yes | Yes | Yes | Yes | Yes | Yes | Yes | N/A | Yes | Yes |
| Pellegrini, 2015 | Yes | Yes | Yes | Yes | Yes | Yes | Yes | Yes | Yes | N/A | Yes | Yes |
| Rabbi, 2015 [1, 2] | Yes | Yes | Yes | Yes | Yes | Yes | Yes | Yes | Yes | N/A | Yes | Yes |
| Thomas, 2015 | Yes | Yes | Yes | Yes | Yes | Yes | Yes | Yes | Yes | N/A | Yes | Yes |
| Ben-Zeev, 2014 | Yes | Yes | Yes | Yes | Yes | Yes | Yes | Yes | Yes | N/A | Yes | Yes |
| Gustafson, 2014 | Yes | Yes | Yes | Yes | Yes | Yes | Yes | Yes | Yes | N/A | Yes | Yes |
| Van Dantzig, 2013 | Yes | Yes | Yes | Yes | Yes | Yes | Yes | Yes | Yes | N/A | Yes | Yes |
| Burns, 2011 | Yes | Yes | Yes | Yes | Yes | Yes | Yes | Yes | Yes | N/A | Yes | Yes |
| Lin, 2011 | Yes | Yes | Yes | Yes | Yes | Yes | Yes | Yes | Yes | N/A | Yes | Yes |

## Appendix S8: Assessment of intervention reporting clarity

| **Included studies** | **Decision points** | **Tailoring variables** | **Intervention options** | **Decision rules** | **Proximal outcomes** | **Distal outcomes** |
| --- | --- | --- | --- | --- | --- | --- |
| Wang, 2023 | Yes | Yes | Yes | Yes | Yes | Yes |
| Yang, 2023 | Yes | Yes | Yes | Yes | Yes | Yes |
| Beres, 2021, 2022 | Yes | Yes | Yes | Not sufficiently reported | Yes | Yes |
| Carlozzi, 2022 | Yes | Yes | Yes | Yes | Yes | Yes |
| Ismail, 2022 | Yes | Yes | Yes | Yes | Yes | Yes |
| Juarascio, 2022 | Yes | Yes | Not sufficiently reported | Yes | Yes | Yes |
| Mair, 2022 | Yes | Yes | Yes | Yes | Yes | Yes |
| Morgiève, 2022 | Yes | Yes | Yes | Yes | Yes | Yes |
| Sizemore, 2022 | Yes | Yes | Yes | Yes | Yes | Yes |
| Walters, 2022 | Yes | Yes | Yes | Yes | Yes | Yes |
| Blevins, 2021 | Yes | Yes | Yes | Yes | Yes | Yes |
| Coughlin, 2021 | Yes | Yes | Yes | Yes | Yes | Yes |
| Fulford, 2021 | Yes | Yes | Yes | Yes | Yes | Yes |
| Gire, 2021 | Yes | Not sufficiently reported | Yes | Yes | Yes | Yes |
| Hawker, 2021 | Yes | Yes | Yes | Yes | Yes | Yes |
| Juarascio, 2021 [1] | Yes | Yes | Yes | Yes | Yes | Yes |
| Juarascio, 2021 [2] | Yes | Yes | Yes | Yes | Yes | Yes |
| Santa Maria, 2021 | Yes | Yes | Yes | Yes | Yes | Yes |
| Wang, 2021 | Yes | Yes | Yes | Yes | Yes | Yes |
| Conroy, 2020 | Yes | Yes | Yes | Yes | Yes | Yes |
| Hébert, 2020 | Yes | Yes | Yes | Yes | Yes | Yes |
| Low, 2020 | Yes | Yes | Yes | Yes | Yes | Yes |
| NeCamp, 2020 | Yes | Yes | Yes | Yes | Yes | Yes |
| Scott, 2020 | Yes | Yes | Yes | Yes | Yes | Yes |
| Shrier, 2020 | Yes | Yes | Yes | Yes | Yes | Yes |
| Valle, 2020 | Yes | Yes | Yes | Yes | Yes | Yes |
| Bartlett Ellis, 2019 | Yes | Yes | Yes | Yes | Yes | Yes |
| Forman, 2019 [1, 2] | Yes | Yes | Yes | Yes | Yes | Yes |
| Hiremath, 2019 | Yes | Yes | Yes | Yes | Yes | Yes |
| Hoeppner, 2019 | Yes | Yes | Not sufficiently reported | Not sufficiently reported | Yes | Yes |
| Klasnja, 2019 | Yes | Yes | Yes | Yes | Yes | Yes |
| Levin, 2019 [1, 2] | Yes | Yes | Yes | Yes | Yes | Yes |
| O’Donnell, 2019 | Yes | Yes | Yes | Yes | Yes | Yes |
| Hébert, 2018 | Yes | Yes | Yes | Yes | Yes | Yes |
| Shrier, 2018, 2014 | Yes | Yes | Yes | Yes | Yes | Yes |
| Van Dantzig, 2018 | Yes | Yes | Yes | Yes | Yes | Yes |
| Attwood, 2017 | Yes | Yes | Yes | Yes | Yes | Yes |
| Leonard, 2017 | Yes | Yes | Yes | Yes | Yes | Yes |
| Businelle, 2016 | Yes | Yes | Yes | Yes | Yes | Yes |
| Ding, 2016 | Yes | Yes | Yes | Yes | Yes | Yes |
| Naughton, 2016 | Yes | Yes | Yes | Yes | Yes | Yes |
| Wenze, 2016 | Yes | Yes | Yes | Yes | Yes | Yes |
| Depp, 2015 | Yes | Yes | Yes | Yes | Yes | Yes |
| Finkelstein, 2015 | Yes | Yes | Yes | Yes | Yes | Yes |
| Gonzalez, Dulin, 2015, 2014 | Yes | Yes | Yes | Yes | Yes | Yes |
| Ingersoll, 2015, 2014 | Yes | Yes | Yes | Yes | Yes | Yes |
| Mundi, 2015 | Yes | Yes | Yes | Not sufficiently reported | Yes | Yes |
| Pellegrini, 2015 | Yes | Yes | Yes | Yes | Yes | Yes |
| Rabbi, 2015 [1, 2] | Yes | Yes | Yes | Yes | Yes | Yes |
| Thomas, 2015 | Yes | Yes | Yes | Yes | Yes | Yes |
| Ben-Zeev, 2014 | Yes | Yes | Not sufficiently reported | Yes | Yes | Yes |
| Gustafson, 2014 | Yes | Yes | Yes | Yes | Yes | Yes |
| van Dantzig, 2013 | Yes | Yes | Yes | Yes | Yes | Yes |
| Burns, 2011 | Yes | Yes | Yes | Yes | Yes | Yes |
| Lin, 2011 | Yes | Yes | Yes | Yes | Yes | Yes |

## Appendix S9: Example of using JITAI reporting checklist

| **Categories** | **Items** | | **Guidance** | **Example (Hawker, 2021)** |
| --- | --- | --- | --- | --- |
| **Brief name**: what is the name of the intervention? | Item 1 | Intervention type | Describe the intervention design as just-in-time adaptive intervention | A just-in-time adaptive intervention called GamblingLess: Curb Your Urge |
| **Why**: why was the intervention developed, and why was the study conducted? | Item 2 | Intervention rationale | Describe any rationale, theory, or goal of the elements essential to the intervention | GamblingLess: Curb Your Urge was developed as an app-delivered intervention within a suite of evidence-based web and mobile CBT and MI programs (GamblingLess) for problem gambling. The intervention was designed to specifically target gambling cravings based on the relapse prevention model and recent evidence that real-time cravings and self-efficacy to resist cravings were the strongest predictors of subsequent gambling behaviour… |
|  | Item 2-1 | Research purposes | Provide a statement of the aims or objectives behind the research being conducted | This study primarily aims to examine the acceptability and feasibility of GamblingLess. The secondary aim of this study is to explore the intervention’s preliminary effectiveness, with the intervention hypothesized to (1) reduce real-time gambling craving intensity from immediately before to immediately after using the intervention and (2) reduce the preintervention levels of gambling symptom severity, gambling cravings, gambling frequency and expenditure, craving self-efficacy, and gambling self-efficacy at postintervention and 1-month follow-up evaluations… |
|  | Item 2-2 | Research design | Outline the systematic plans used to conduct the research | This study was a single-arm, 5-week acceptability and feasibility trial (1-week baseline and 4-week intervention periods) involving EMAs delivered 3 times daily and web-based evaluations at baseline, postintervention, and a 1-month follow-up… |
|  | Item 2-3 | Target behaviours | State the behaviour(s) evaluated in the intervention | Gambling behaviour |
|  | Item 2-4 | Target population | State the participants characteristics targeted by the intervention | Adults with gambling problems |
| **What**: what intervention materials and procedures were undertaken? | Item 3 | Intervention materials | Describe any physical or informational materials used in the intervention, including those provided to participants or used in intervention delivery or in training of intervention providers. Provide information on where the materials can be accessed (e.g. online appendix, URL) | In this trial, GamblingLess: Curb Your Urge consisted of a 4-week EMI delivered by an existing smartphone app platform (MetricWire). The intervention comprised 12 urge-curbing tips and activities (eg, About My Urge, Delay and Distract, and Urge Surfing)… |
|  | Item 4 | Intervention procedures | Describe each of the procedures, activities, and/or processes used in the intervention, including any enabling or support activities | Study advertisements directed participants to the web-based baseline questionnaire, which was preceded by the provision of study information (eg, what participation involves and a description of the app intervention) followed by participant consent. In the baseline questionnaire, participants provided their contact details, which were used to email participants an instruction manual for downloading and using the app (eg, submitting EMAs and accessing the intervention content). Once enrolled, participants completed a 1-week EMA-only baseline period, followed by a 4-week intervention period (ie, EMA or EMI and 24×7 on-demand access to the intervention content). At the end of the intervention period, participants could no longer access the intervention. Participants were emailed a link to the web-based postintervention and 1-month follow-up questionnaires and, if required, were emailed up to 3 times to encourage questionnaire completion… |
| **Who provided**: Who or what tools were used to provide the intervention? | Item 5 | Intervention providers | For each category of intervention provider (e.g. psychologist, nursing assistant), describe their expertise, background and any specific training given. If the provider is a computerised system, describe relevant information of the system | GamblingLess: Curb Your Urge consisted of a 4-week EMI delivered by an existing smartphone app platform (MetricWire)… |
| **How**: how the intervention was delivered, and how user data and outcomes were captured, processed, and evaluated? | Item 6 | Delivery mode | Describe the modes of delivery (e.g. face-to-face or by some other mechanism, such as internet or telephone) of the intervention and whether it was provided individually or in a group | Through a smartphone app |
|  | Item 6-1 | Data capturing methods | Detail the techniques and tools used to collect information for tailoring the intervention, including both self-report and passive monitoring | The app administered a brief EMA at random times during three prespecified periods (9 AM to noon, 1 PM-4 PM, and 5 PM-8 PM) via push notifications. The EMA comprised five coreitems to measure gambling episodes (since the last EMA), gambling cravings (current and since the last EMA), gambling self-efficacy (current confidence in ability to limit or stop gambling), and craving self-efficacy (current confidence in the ability to resist cravings) and up to an additional seven items depending on core item responses. These additional items were included to gain descriptive data about gambling cravings (eg, duration, frequency) that are largely absent in the corresponding empirical literature. The EMA took approximately 1-2 minutes to complete, depending on the pattern of responses |
|  | Item 6-2 | Data processing methods | Provide details on the specific techniques used for processing data, including their sources, the development process, and the intended purposes and contexts for use. This description should clarify how the data was prepared and transformed prior to its use in informing intervention delivery | No information |
|  | Item 6-3 | Outcome measures | Detail the metrics or standardised tools used to assess the study objectives, ideally breaking down into subjective and objective measurements | Feasibility was also assessed using several app use metrics, including EMA compliance and EMI compliance. Feasibility was also assessed via intervention use more generally…  Acceptability was assessed via (1) helpfulness of each urge-curbing tip or activity, and the relevance and burden of EMA items, via separate 11-point Visual Analogue Scales (VAS) from 0 (not helpful or relevant or burdensome) to 10 (very helpful or relevant or burdensome). (2) satisfaction with the intervention via the 3-item Client-Satisfaction Questionnaire-3 (CSQ-3; total score range 3-12). (3) impact of the intervention on participants’ awareness, knowledge, attitude, intention to change, help-seeking behavior, and behavior change in relation to gambling cravings via the 6-item App-Specific subscale of the Mobile App Rating Scale (MARS), whereby the mean item score of 3 (range 1-5) indicates minimum acceptability. (4) a series of open-ended items assessing suggested improvements for any tip or activity that participants rated a 5 or less (out of 10) for helpfulness (How could [tip/activity] be improved?), any technical issues (Please comment on any technical issues experienced.), and general feedback about the app intervention (Do you have any other comments or feedback?)… |
| **Where**: where was the intervention provided, and how were ethical issues, data protection and sharing methods handled? | Item 7 | Delivery setting | Describe the type(s) of location(s) where the intervention occurred, including any necessary infrastructure or relevant features | User’s everyday environments |
|  | Item 7-1 | Research ethics | Provide the ethical approvals and permission obtained, including the types and sources of the approvals | This study received ethics approval from the Deakin University Human Research Ethics Committee (Ethics ID: 2019-030). |
|  | Item 7-2 | Data protection methods | Describe the technical and administrative measures implemented to ensure the security and privacy of remote data collection and monitoring, including information on the regulations or guidelines followed | No information |
|  | Item 7-3 | Data sharing policies | Describe the policies and regulations followed for data sharing between researchers and participants, including any involvement of third parties | No information |
| **When and How** **much**: when and how often was the intervention delivered, and what was the duration and dosage of the intervention? | Item 8 | Intervention dosage | Describe the number of times the intervention was delivered and over what period of time including the number of sessions, their schedule, and their duration, intensity or dose | The app’s EMI feature involved an automatic recommendation to use any urge-curbing tip or activity at the end of any EMA in which participants reported that they had a current craving (ie, responded Yes to EMA item 1)… |
| **Tailoring**: was the intervention tailored, titrated or adapted, and if so, how? | Item 9 | Tailoring | If the intervention was planned to be personalised, titrated or adapted, describe any rationale, theory, or goal essential to the tailoring variables | Similar to other app-delivered EMIs, the intervention provides a hybrid model, comprising a push intervention, whereby the app recommends an intervention at times of identified need, and a pull intervention, whereby consumers can access an intervention on demand, whenever and wherever they need it, without needing to complete an EMA. The intervention was intended for use as a stand-alone or adjunctive treatment or relapse prevention tool… |
|  | Item 9-1 | Decision points | Explain the timing when an intervention decision is made, including frequency, time of the day, duration of the assessment process | The app administered a brief EMA at random times during three prespecified periods (9 AM to noon, 1 PM-4 PM, and 5 PM-8 PM) via push notifications. |
|  | Item 9-2 | Tailoring variables | Detail the sources of data used in making an intervention decision, including both self-reported and passively monitored information | The EMA comprised five core items to measure gambling episodes (since the last EMA), gambling cravings (current and since the last EMA), gambling self-efficacy (current confidence in ability to limit or stop gambling), and craving self-efficacy (current confidence in the ability to resist cravings) and up to an additional seven items depending on core item responses. These additional items were included to gain descriptive data about gambling cravings (eg, duration, frequency) that are largely absent in the corresponding empirical literature… |
|  | Item 9-3 | Intervention options | Describe the various types of support offered in tailored scenarios, organising them into clear and understandable categories for better comprehension | The app’s EMI feature involved an automatic recommendation to use any urge-curbing tip or activity at the end of any EMA in which participants reported that they had a current craving (ie, responded Yes to EMA item 1. The program was also available 24×7 for use on demand. Whenever participants used a tip or activity (regardless of whether use was recommended or on demand), they were asked to rate the intensity of their craving to gamble from 0 (mild) to 10 (severe) immediately before and after using the tip or activity… |
|  | Item 9-4 | Decision rules | Detail the metrics and criteria used to determine the choice of intervention and its timing, providing a diagram to illustrate the decision-making process if possible | At the end of any EMA in which participants reported that they had a current craving (ie, responded Yes to EMA item 1) |
|  | Item 9-5 | Proximal outcomes | Describe the target behaviour that the intervention aims to change, and how they are related to the overall desired outcome | Gambling urge, craving intensity |
|  | Item 9-6 | Distal outcomes | State the ultimate goal or primary clinical outcome of the intervention | Gambling episodes |
| **Modification**: was the intervention procedure modified at any point, and if so, how? | Item 10 | Intervention modification | If the intervention was modified during the course of the study, describe the changes (what, why, when, and how) | N/A |
| **How well**: was the adherence assessed, and if so, what methods were used to evaluate it and how well did it perform? | Item 11 | Adherence plans | If intervention adherence or fidelity was assessed, describe how and by whom, and if any strategies were used to maintain or improve fidelity, describe them | Adherence was assessed via participant recruitment at baseline and retention at postintervention and follow-up evaluations. EMA compliance was measured as the rate at which participants completed the EMA during the baseline and intervention periods. EMI compliance was measured as the rate at which participants completed any intervention content within 60 minutes of receiving an EMI recommendation to use the intervention because they reported a current craving to gamble during an EMA… |
|  | Item 12 | Adherence results | If intervention adherence or fidelity was assessed, describe the extent to which the intervention was delivered as planned | Over the 1-month recruitment period, 56 gamblers consented to participate in this study. Of these, 36 gamblers completed the baseline measures, 14 gamblers did not complete any baseline measures, and 6 gamblers completed only the baseline demographic measures. Of the 36 participants who completed the baseline measures, 9 participants did not download the app, 1 participant downloaded the app but did not use it, and 4 participants did not use the app beyond the first week of the study and did not complete the postintervention evaluation. The remaining 22 participants used the app and completed the postintervention evaluation, with 21 of these participants completing the 1-month follow-up evaluation. Of the 36 participants who completed baseline measures, 61% (22/36) completed postintervention evaluations and 58% (21/36) completed follow-up evaluations. There were no significant differences in any baseline variable between participants who did or did not complete the postintervention evaluation. The EMAs had a compliance rate of 68% in the baseline period (mean 14.27, SD 5.68 out of 21 EMAs), 47% in the intervention period (mean 54.05, SD 33.37 out of 84 EMAs), and 51% overall (mean 54.05, SD 33.37 out of 105 EMAs). The EMI compliance rate was 15%, as participants used an intervention 13 out of the 87 times that they were recommended to do so based on craving occurrence… |
